# Nucleocytosolic Depletion of the Energy Metabolite Acetyl-Coenzyme A Stimulates Autophagy and Prolongs Lifespan

**DOI:** 10.1016/j.cmet.2014.02.010

**Published:** 2014-03-04

**Authors:** Tobias Eisenberg, Sabrina Schroeder, Aleksandra Andryushkova, Tobias Pendl, Victoria Küttner, Anuradha Bhukel, Guillermo Mariño, Federico Pietrocola, Alexandra Harger, Andreas Zimmermann, Tarek Moustafa, Adrian Sprenger, Evelyne Jany, Sabrina Büttner, Didac Carmona-Gutierrez, Christoph Ruckenstuhl, Julia Ring, Wieland Reichelt, Katharina Schimmel, Tina Leeb, Claudia Moser, Stefanie Schatz, Lars-Peter Kamolz, Christoph Magnes, Frank Sinner, Simon Sedej, Kai-Uwe Fröhlich, Gabor Juhasz, Thomas R. Pieber, Jörn Dengjel, Stephan J. Sigrist, Guido Kroemer, Frank Madeo

**Affiliations:** 1 Institute of Molecular Biosciences, University of Graz, Humboldtstrasse 50, 8010 Graz, Austria; 2 Freiburg Institute for Advanced Studies (FRIAS), University of Freiburg, Albertstrasse 19, 79104 Freiburg, Germany; 3 Department of Dermatology, University Freiburg Medical Center, Hauptstrasse 7, 79104 Freiburg, Germany; 4 Institute for Biology/Genetics, Freie Universität, Takustraße 6, 14195 Berlin, Germany; 5 NeuroCure, Charité, Charitéplatz 1, 10117 Berlin, Germany; 6 INSERM U848, Pavillon de Recherche 1, 94805 Villejuif, France; 7 Metabolomics and Cell Biology Platforms, Institut Gustave Roussy, Pavillon de Recherche 1, 94805 Villejuif, France; 8 Université Paris Sud, Faculté de Médecine, 63 Rue Gabriel Péri, 94270 Le Kremlin Bicêtre, France; 9 Division of Endocrinology and Metabolism, Department of Internal Medicine, Medical University of Graz, Auenbruggerplatz 15, 8036 Graz, Austria; 10 Division of Plastic, Aesthetic and Reconstructive Surgery, Department of Surgery, Medical University Graz, Auenbruggerplatz 29, 8036 Graz, Austria; 11 HEALTH–Institute for Biomedicine and Health Sciences, Joanneum Research Forschungsgesellschaft m.b.H., Leonhardstraße 59, 8010 Graz, Austria; 12 Department of Cardiology, Medical University of Graz, Auenbruggerplatz 15, 8036 Austria; 13 Department of Anatomy, Cell, and Developmental Biology, Eotvos Lorand University, Egyetem tér 1–3, 1053 Budapest, Hungary; 14 Equipe 11 Labellisée Ligue Contre le Cancer, INSERM U1138, Centre de Recherche des Cordeliers, 15 Rue de l’École de Médecine, 75006 Paris, France; 15 Pôle de Biologie, Hôpital Européen Georges Pompidou, AP-HP, 20 Rue Leblanc, 75908 Paris, France; 16 Université Paris Descartes, Sorbonne Paris Cité, 12 Rue de l’École de Médecine, 75006 Paris, France

## Abstract

Healthy aging depends on removal of damaged cellular material that is in part mediated by autophagy. The nutritional status of cells affects both aging and autophagy through as-yet-elusive metabolic circuitries. Here, we show that nucleocytosolic acetyl-coenzyme A (AcCoA) production is a metabolic repressor of autophagy during aging in yeast. Blocking the mitochondrial route to AcCoA by deletion of the CoA-transferase *ACH1* caused cytosolic accumulation of the AcCoA precursor acetate. This led to hyperactivation of nucleocytosolic AcCoA-synthetase Acs2p, triggering histone acetylation, repression of autophagy genes, and an age-dependent defect in autophagic flux, culminating in a reduced lifespan. Inhibition of nutrient signaling failed to restore, while simultaneous knockdown of *ACS2* reinstated, autophagy and survival of *ach1* mutant. Brain-specific knockdown of *Drosophila* AcCoA synthetase was sufficient to enhance autophagic protein clearance and prolong lifespan. Since AcCoA integrates various nutrition pathways, our findings may explain diet-dependent lifespan and autophagy regulation.

## Highlights

• Acetyl-CoA (AcCoA) metabolism regulates autophagy during aging
• Autophagy regulation by AcCoA metabolism acts downstream of nutrient signaling
• Brain-specific knockdown of *Drosophila AcCoA synthetase* prolongs lifespan
• Histone point mutations permanently activate autophagy during aging

## Graphical Abstract

**Figure:**
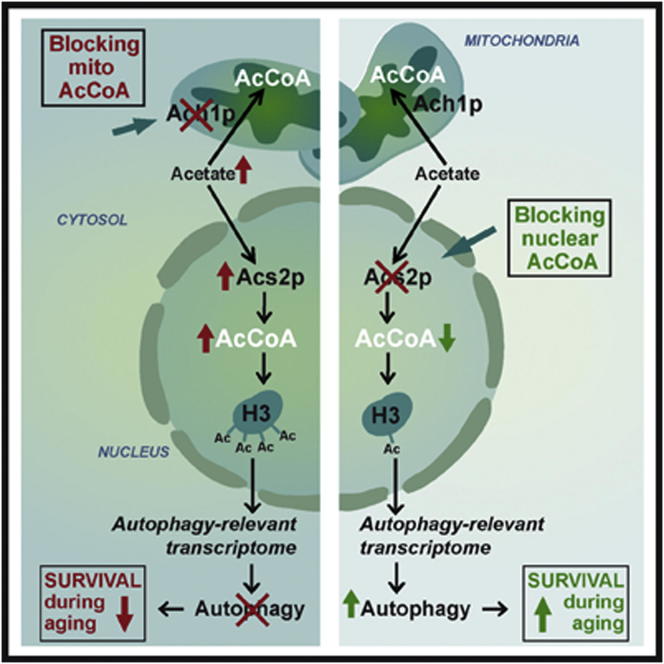

## Teaser

Autophagy plays a crucial role in healthy aging. By blocking mitochondrial AcCoA production, Eisenberg et al. show that accumulation of nucleocytosolic AcCoA inhibits autophagy and reduces lifespan through a conserved epigenetic mechanism involving histone acetylation of specific autophagy genes in yeast and flies.

## Introduction

Aging is accompanied by accumulation of cellular damage, changes in the repair and detoxification processes, and a shifting homeostatic balance in conflicting lethal and vital signaling programs (Madeo et al., 2010a; Rubinstein and Kimchi, 2012). Macroautophagy (hereafter referred to as “autophagy”) is a bulk degradation pathway in which parts of the cytosol or cytoplasmic organelles are encapsulated into double-membraned vesicles, so-called autophagosomes, which ultimately fuse with vacuoles/lysosomes where the cytoplasmic material becomes degraded (He and Klionsky, 2009). Autophagy plays a major role in the maintenance of cellular homeostasis, recycling energy reserves in the context of dwindling external resources and contributing to the removal of damaged organelles and potentially harmful protein aggregates (Chen and White, 2011; Kroemer et al., 2010; Mizushima and Komatsu, 2011). Autophagy therefore has emerged as a pivotal cellular process that can delay the pathogenic manifestations of aging and age-associated disease (Gelino and Hansen, 2012; Madeo et al., 2010a; Rubinsztein et al., 2011). While regulation of autophagy during short-term induction conditions (up to several hours) is well-investigated, understanding the long-term regulation of autophagy during the process of aging remains a challenge.

Autophagy is profoundly influenced by nutrient-responsive kinases, including the target of rapamycin (TOR), protein kinase A (PKA), and AMP-activated protein kinase (AMPK), all of which are also well-known regulators of aging and lifespan (Fontana et al., 2010; He and Klionsky, 2009; Kenyon, 2010). However, recent studies suggest that protein acetylation, a process that is lately recognized as a posttranslational modification that rivals phosphorylation in importance (Choudhary et al., 2009; Guan and Xiong, 2011; Sadoul et al., 2011), may also regulate autophagy at targets distinct from that of known kinase regulators (Geeraert et al., 2010; Lee et al., 2008; Morselli et al., 2011; Xie et al., 2010; Yi et al., 2012). While acetylation of ATG3 and of tubulin is required for autophagy (Geeraert et al., 2010; Xie et al., 2010; Yi et al., 2012), ATG7 must be deacetylated by sirtuin 1 for the initiation of starvation-induced autophagy (Lee et al., 2008). Autophagy is in principle a cytoplasmic process that does not require a nuclear-localized transcriptional response for short-term activity, as demonstrated by the ability of cytoplasts (i.e., enucleated cells) to undergo starvation-induced autophagy (Morselli et al., 2011). Nevertheless, regulation of age-associated autophagy may depend on epigenetic processes (including that of histone acetylation) as well as on specific transcription factors such as FoxO3 that transactivate the autophagy-relevant transcriptome necessary for enduring autophagic activity that exceeds beyond a few hours (Eisenberg et al., 2009; Settembre et al., 2011; Zhao et al., 2007). Acetylation at lysine 16 of histone H4 has only recently been demonstrated to determine the outcome of autophagy (i.e., cytotoxic versus protective), possibly by influencing the transcriptional status of *ATG* genes (Füllgrabe et al., 2013), and the Ume6p transcription factor regulates the size of autophagosomes through control of *ATG8* expression levels (Bartholomew et al., 2012).

Acetyl-coenzyme A (AcCoA) serves as an acetyl-group donor for protein and histone acetylation in eukaryotic cells and at the same time represents a central metabolite of cellular energy metabolism. It has been proposed that metabolism may connect to various cellular functions by modulating intracellular metabolites (including that of AcCoA), which in turn act as cofactors for posttranslational modifications affecting enzyme function or epigenetic status of the chromatin (Kaelin and McKnight, 2013; Lu and Thompson, 2012). Given the increasingly recognized role of protein acetylation as well as histone acetylation in the regulation of autophagy, we therefore aimed at evaluating the hypothesis that AcCoA biosynthetic availability would affect autophagy in a long-term context, in chronological aging.

For more than a decade now, budding yeast has significantly contributed to our understanding of aging, unraveling mechanisms of cellular senescence and age-induced death of postmitotic cells in a model of chronological aging (Kaeberlein, 2010; Longo et al., 2012). In budding yeast, de novo synthesis of AcCoA is facilitated by two distinguishable metabolic routes, namely the mitochondrial (*ACS1*-, *ACH1*-, or *MPC1*-dependent) versus the nucleocytosolic (*ACS2*-dependent) pathways (Bricker et al., 2012; Fleck and Brock, 2009; Takahashi et al., 2006). Based on these premises, we determined the autophagy-modulatory effects of manipulating the de novo biosynthetic pathways of AcCoA generation in a model of chronological aging.

## Results

### Knockdown of *ACS2* Is Sufficient to Promote Autophagy during Aging

**Figure 1:**
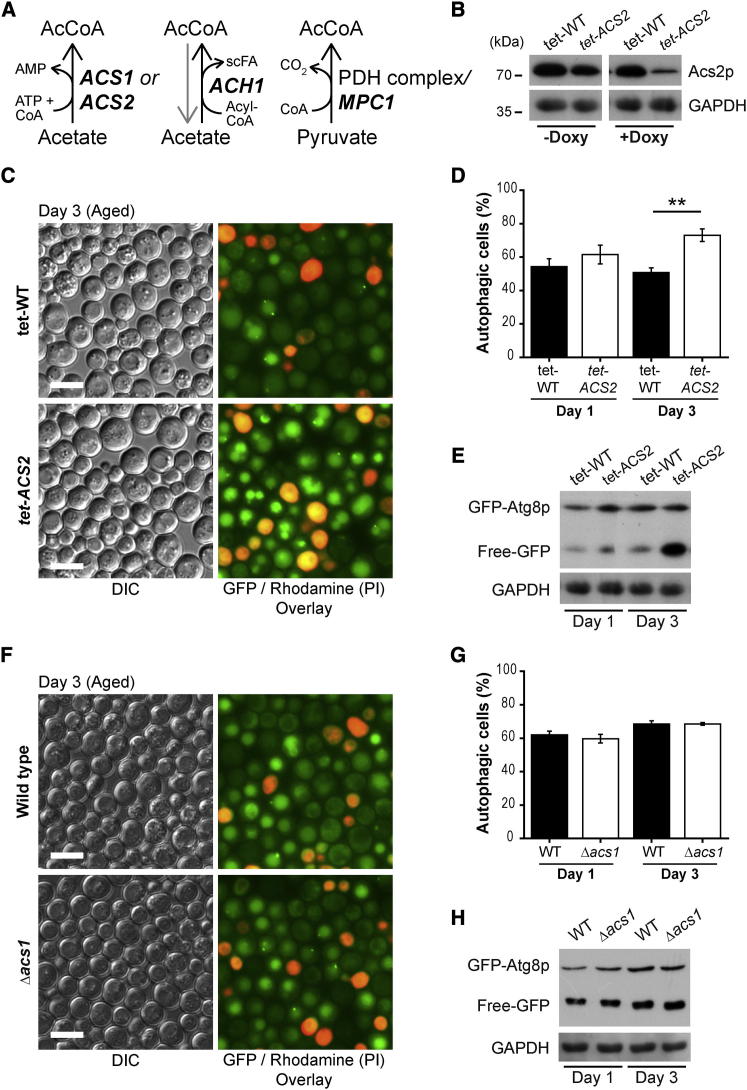
*ACS2* Depletion Ameliorates Age-Associated Autophagy in Yeast (A) Scheme of known major acetyl-CoA (AcCoA)-producing enzymes (bold characters) modulated in this study. (B) Representative immunoblot of GFP-Atg8p expressing yeast. Wild-type cells (tet-WT) were compared to strains carrying a doxycycline-repressible tet-O7 promoter controlling *ACS2* transcription (*tet-ACS2*). Cells were grown in SC 2% glucose medium for 24 hr (day 1 of aging) in the presence (+Doxy) or absence (−Doxy) of 1 ng/ml doxycycline to induce knockdown of *ACS2* (see also Figure S1). (C) Fluorescence microscopy of GFP-Atg8p expressing (under control of its natural pATG8 promoter) wild-type (tet-WT) and *ACS2* knockdown (*tet-ACS*) cells grown in the presence of 1 ng/ml doxycycline (as shown in A) and chronologically aged for 3 days. Propidium iodide (PI) counterstaining served to visualize dead cells. Scale bars represent 5 μm. (D) Quantification of cells depicted in (C) (day 3) and of young (day 1) cells with 150–300 counts (blinded) for each replicate. Autophagic cells were defined as cells with clear vacuolar GFP fluorescence. Data represent means ± SEM (n = 4). ^∗∗^p < 0.01. (E) Representative immunoblot analysis of day 1 (young controls) and day 3 (aged) cells shown in (C) and (D) using anti-GFP and anti-GAPDH (loading control) antibodies to detect “free-GFP” indicative of autophagic flux. (F–H) Representative micrographs (F), respective quantification (G), and immunoblot analysis (H) of wild-type (WT) and *ACS1*-deleted (Δ*acs1*) yeast aged to day 3 compared to young (day 1) cells expressing GFP-Atg8p chimera as in (C)–(E). Data represent means ± SEM (n = 4).

To test if de novo AcCoA biosynthesis from acetate or pyruvate modulated the autophagic response during cellular aging, we screened deletion or knockdown mutants of genes encoding enzymes involved in AcCoA formation (see scheme in Figure 1A). To this end, we first assessed the potential influence of the AcCoA synthetases Acs1p and Acs2p that generate AcCoA through the ATP-dependent condensation of acetate and coenzyme A (CoA). We optimized the experimental conditions for knockdown of the growth essential *ACS2* gene using a doxycycline-repressible tetO7-promoter (*tet-ACS2*) so that cells would manifest a significant depletion of the Acs2 protein (Figure 1B) yet only partially reduce their growth during the logarithmic phase, yielding a rather modest reduction of cell densities upon entry into the stationary phase as compared to wild-type controls (tet-WT) (1 ng/ml doxycycline, Figure S1, available online). Knockdown of *ACS2* led to a strong induction of autophagy upon chronological aging, as monitored by assessing the autophagy-dependent translocation of a cytosolic green fluorescent protein (GFP)-Atg8p fusion to the vacuole (Figures 1C and 1D). Both the number of autophagic cells, defined as cells that displayed clearly vacuolar localization of GFP (Figure 1C; quantified in Figure 1D), and vacuolar GFP intensities increased upon knockdown of *ACS2*. To strengthen these findings, we used a complementary assay based on the immunoblot detection of *liberated* GFP that is generated upon the autophagy-associated delivery of GFP-Atg8p to the vacuole and subsequent proteolytic removal of Atg8p (Klionsky et al., 2007). This *GFP liberation assay* demonstrated enhanced autophagic flux with active vacuolar proteolysis in aged *tet-ACS2* cells compared to its wild-type (tet-WT) counterpart (Figure 1E). In contrast to knockdown of *ACS2*, age-associated autophagy was not affected by deletion of *ACS1* (Figures 1F–1H).

Thus, while deletion of *ACS1*, the gene which is known to be suppressed by glucose (van den Berg et al., 1996), was dispensable for age-dependent autophagic activity, knockdown of *ACS2*—the crucial gene for AcCoA production on glucose conditions (Takahashi et al., 2006)—was sufficient to enhance the autophagic response to aging.

### Mitochondrial AcCoA Production Pathways Are Required for Age-Associated Autophagy

**Figure 2:**
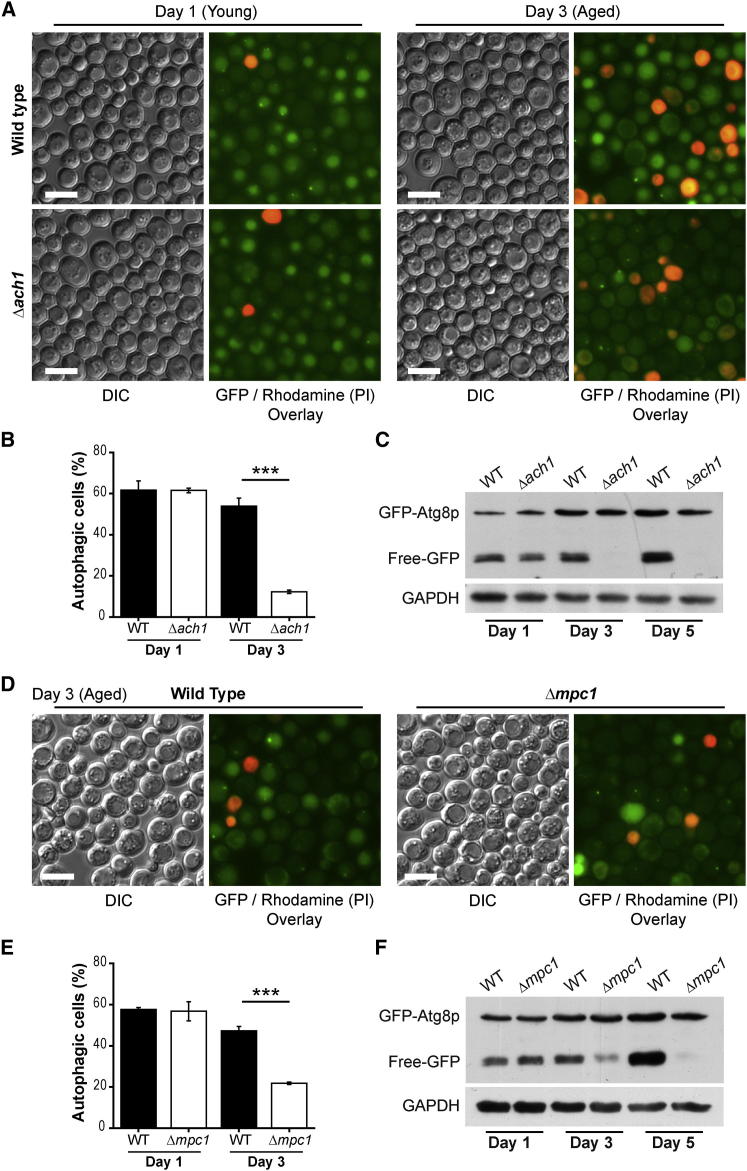
Mitochondrial AcCoA Production by Ach1p- or Mpc1p-Associated Pathways Is Required for Autophagy during Aging (A) Fluorescence microscopy of GFP-Atg8p expressing wild-type (WT) and *ACH1*-deleted (Δ*ach1*) yeast grown to day 1 (young) and aged for 3 days in SC 2% glucose medium; propidium iodide (PI) counterstaining served to visualize dead cells. Scale bars represent 5 μm. (B) Quantification of cells depicted in (A) with 150–300 counts (blinded) for each replicate. Autophagic cells were defined as cells with clear vacuolar GFP fluorescence. Data represent means ± SEM (n = 4). ^∗∗∗^p < 0.001. (C) Representative immunoblot analysis of cells shown in (A) and further aged to day 5 using anti-GFP and anti-GAPDH (loading control) antibodies to detect “free-GFP” indicative of autophagic flux (see also Figure S2). (D–F) Representative micrographs (D), respective quantification (E), and immunoblot analysis (F) of wild-type (WT) and *MPC1* deleted (Δ*mpc1*) yeast expressing GFP-Atg8p chimera as in (A)–(C) and aged to indicated time points. Data represent means ± SEM (n = 4). ^∗∗∗^p < 0.001.

We next asked if blocking AcCoA synthesis through Ach1p, which transfers CoA from short-chain Acyl-CoAs to acetate generating AcCoA (Fleck and Brock, 2009), would also affect the autophagic response of aging cells. Remarkably, deletion of *ACH1* completely blocked the autophagic activity after 3 days of aging, while young (day 1) cultures presented comparable levels of autophagy-competent cells (manifesting vacuolar localization of GFP-Atg8p as well as *GFP liberation* activity) in both wild-type and *ach1* mutant (Figures 2A–2C). In addition, we assessed the autophagic flux by measuring vacuolar alkaline phosphatase (vac. ALP) activity using yeast strains carrying an engineered form of *PHO8* (*pho8ΔN60*), encoding a cytosolically localized phosphatase, which becomes activated through autophagic delivery to the vacuole (Noda and Klionsky, 2008). While showing comparable levels of vac. ALP activity early during aging, *ach1* mutant cells developed a strong defect in vac. ALP activity after 3 days (>72 hr) of aging (Figure S2A). We also measured cell stress by assessing the fraction of cells staining positive upon incubation with dihydroethidine (DHE), which is converted into fluorescent ethidium in ROS-overproducing cells but also gives rise to strong fluorescence in cells that have lost their plasma membrane integrity. This assay revealed that Δ*ach1* cells lost their autophagic capacity before actual cell stress or death occurred (>88 hr; Figure S2A). These observations rule out that the autophagy defect was simply a result of the previously reported increase in cell death of *ach1* mutant cells (Orlandi et al., 2012). Since Ach1p has been suggested to function primarily in mitochondria (Fleck and Brock, 2009), we next assessed whether block of AcCoA production through mitochondrial pyruvate dehydrogenase (PDH) pathway would also impair autophagy of aging yeast. Deletion of *MPC1*, a recently identified transporter of pyruvate into mitochondria crucial for PDH-derived AcCoA formation (Bricker et al., 2012), indeed caused a comparable defect of age-associated autophagy as observed for Δ*ach1* (Figures 2D–2F). The combined deletion of *ACH1* and *MPC1* led to similar kinetics of the age-dependent loss of autophagy (Figure S2B). Together, this strongly suggests that neither the specific CoA-transferase function of Ach1p nor mitochondrial pyruvate supply by Mpc1p was specifically responsible for the autophagic deficiency of *ach1* or *mpc1* mutant cells. Our data demonstrate a strict requirement of mitochondrial AcCoA production pathways for maintaining autophagic flux during aging.

### Mitochondrial AcCoA Biosynthesis Pathways Are Required for Healthy Aging

**Figure 3:**
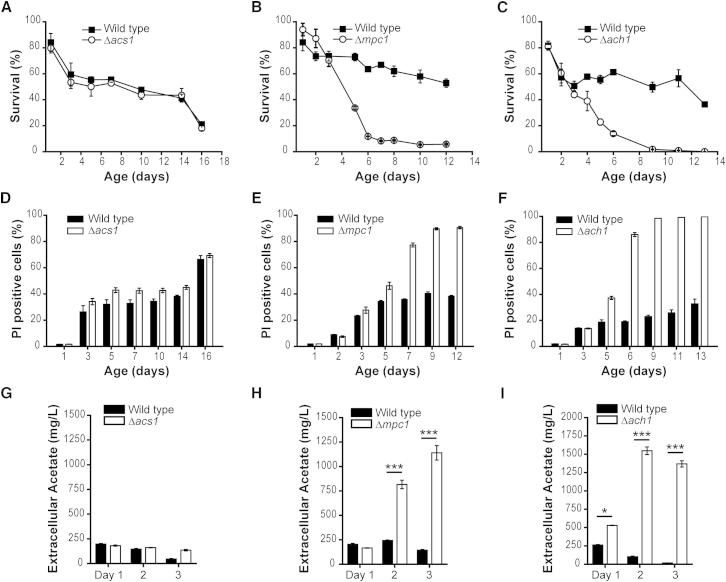
Deletion of *ACH1* or *MPC1* Shortens Chronological Lifespan and Leads to Excess Release of Cellular Acetate (A–C) Chronological lifespan (CLS) analyses in SC 2% glucose medium of wild-type (WT) cells compared to Δ*acs1* (A), Δ*mpc1* (B), or Δ*ach1* (C) cells (see also Figure S3). Survival was determined by colony-forming capacity (clonogenicity). Data represent means ± SEM (n = 4) of a representative aging experiment. (D–F) Propidium iodide (PI)-positive cells analyzed by flow cytometry to quantify age-induced cell death of experiments shown in (A)–(C). Data represent means ± SEM (n = 4) (see also Figure S4). (G–I) Extracellular acetate assessed from crude culture supernatants obtained at indicated time points of experiments shown in (A)–(C). Data represent means ± SEM (n = 4). ^∗^p < 0.05 and ^∗∗∗^p < 0.001.

We next asked if the repression of autophagy induced by blockade of the mitochondrial route to AcCoA biosynthesis would culminate in increased cell death and a reduced lifespan. While deletion or knockdown of *ACS1* or *ACS2*, respectively, barely affected yeast survival during chronological aging as measured by clonogenic assays (Figures 3A and S3A–S3D), deletion of either *ACH1* or *MPC1* dramatically reduced lifespan (Figures 3B and 3C). The frequency of cell death (as determined by propidium iodide-positive cells and further characterized to be of apoptotic and secondary necrotic morphology, see Figure S3I and respective legend for details) consistently increased in short-lived mutant conditions (Δ*ach1* and Δ*mpc1*) compared to wild-type cells and remained largely unchanged upon deletion or knockdown of *ACS1* or *ACS2*, respectively (Figures 3D–3F, S3E, and S3F). Similar to the comparable kinetics of age-associated autophagy impairment, the combined deletion of *ACH1* and *MPC1* showed an epistatic effect on lifespan and age-induced cell death (Figures S3G and S3H). Thus, the consequences of these mutations on lifespan and cell death correlate with the (in)ability to induce a permanently activated autophagy response during aging.

Accumulating extracellular acetate upon deletion of *ACH1* (Fleck and Brock, 2009; Orlandi et al., 2012) has been suggested to trigger the death of *ach1* mutant cells (Orlandi et al., 2012). Accordingly, both *ach1* and *mpc1* mutant cells exhibited an age-dependent and comparable increase in extracellular acetate (Figures 3H and 3I). Deletion of *ACS1* instead entailed rather minor changes in extracellular acetate concentration (Figure 3G), consistent with its lack of an effect on lifespan or autophagic activity.

### Deletion of *MPC1* or *ACH1* Causes Upregulation of the Nucleocytosolic, Acs2p-Mediated Pathway of AcCoA Production

Excess acetate released from cells most likely reflects an overflow of acetate within the cytosol, which may fuel and trigger a cytosolic acetate-metabolizing pathway. Since Acs2p, the nucleocytosolic acetate-converting AcCoA synthetase, appeared limiting for autophagy (remember knockdown provoked autophagy during aging; compare to Figure 1), we hypothesized that upregulation of the Acs2p pathway would mechanistically explain the autophagy defect of *ach1* and *mpc1* mutant cells.

**Figure 4:**
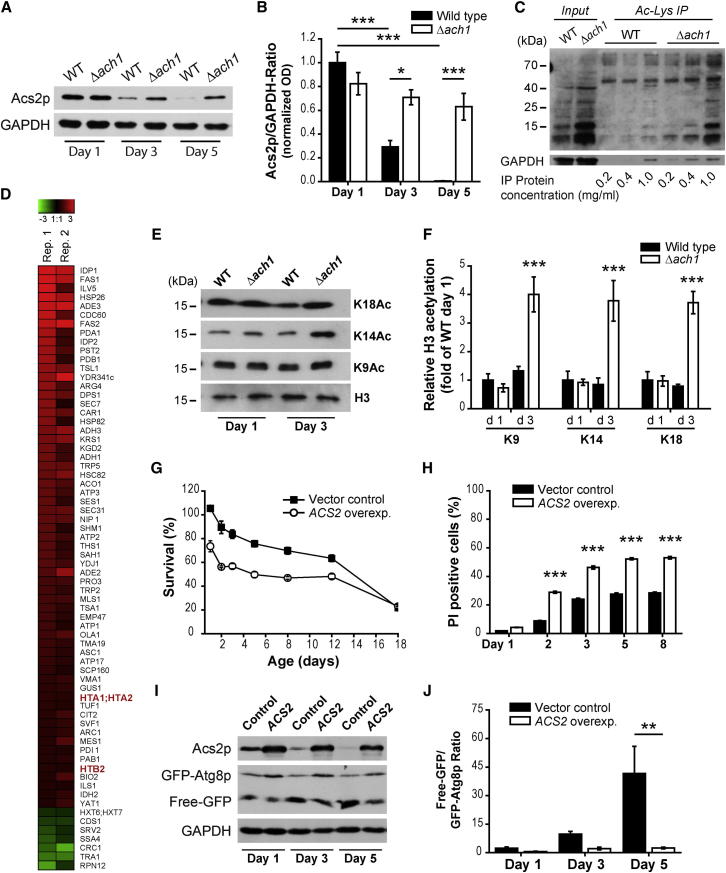
Deletion of *ACH1* or MPC1 Results in Upregulation of the Nucleocytosolic Acs2p Pathway, Causing Histone Hyperacetylation (A and B) Representative immunoblot (A) and densitometric quantification expressed as normalized Acs2p/GAPDH ratios (B) of protein extracts from wild-type (WT) and Δ*ach1* yeast chronologically aged to indicated time points in SC 2% glucose medium (see also Figure S4). (C) Representative immunoblot analysis of 3-day-old WT and Δ*ach1* cells similar to (A) using pan-acetyl-lysine antibodies. Crude protein extracts (*Input*) obtained in the presence of histone deacetylase and sirtuin inhibitors (see Supplemental Experimental Procedures) were subjected to immunoprecipitation (IP) at indicated protein concentrations using pan-acetyl-lysine antibodies to enrich acetylated proteins (*Ac-Lys IP*). GAPDH served as loading control. (D) Heatmap of protein ratios (Δ*ach1* versus WT, log2 scale) corresponding to indicated genes. Data were obtained from mass spectrometric analyses of acetylated proteins enriched by IP similar to (C, 1 mg/ml protein). Extracts from stable-isotope (SILAC)-labeled cells were mixed prior to IP (prelysate), and mean SILAC-protein ratios after IP were normalized to ratios of prelysates to correct for changes in general protein abundance. Two biological replicates were performed (Rep. 1 and Rep. 2) with a regression coefficient of 0.76 of observed protein ratios. Heatmap represents identified proteins with reproducible 1.5-fold upregulation (red color) or downregulation (green color). (E and F) Representative immunoblots of whole cell acid extracts of wild-type (WT) and Δ*ach1* (E) cells chronologically aged to designated time points. Blots were probed with antibodies against total histone H3 (loading control) or H3 acetylated lysines (K9Ac, K14Ac, K18Ac). Densitometric quantification (F) of relative acetylation was calculated as Ac-K/total H3 ratios normalized to ratios of WT at day 1. Data represent means ± SEM (n = 7–8) (see also Figure S5). (G and H) Survival analyses in SC 1.25% galactose/0.75% glucose medium of wild-type cells ectopically overexpressing *ACS2* (*ACS2 overexp*.) compared to vector control cells (Vector control). Survival (I) was determined by colony-forming capacity (clonogenicity) and cell death (J) assessed by propidium iodide (PI)-positive cells analyzed by flow cytometry. Data represent means ± SEM (n = 4) of a representative aging experiment. (I and J) Autophagic flux determination by vacuolar protease-dependent GFP liberation after *ACS2* overexpression compared to empty vector controls (Control) similar to (G) and (H). Representative immunoblots at indicated time points (G) and quantification by densitometry of free-GFP/GFP-Atg8p signal ratios (H) are shown. Data represent means ± SEM (n = 4). ^∗^p < 0.05, ^∗∗^p < 0.01, and ^∗∗∗^p < 0.001.

To test this hypothesis, we first analyzed the protein level of Acs2p in these mutants. While the level of Acs2p decreased during aging in wild-type cells, deletion of *ACH1* or *MPC1* clearly increased the relative amount of Acs2p compared to wild-type cells, suggesting an upregulation of the nucleocytosolic AcCoA pathway under both conditions (Figures 4A, 4B, S4A, and S4B). Interestingly, the sole addition of acetate to aging cultures was similarly efficient in increasing Acs2p protein levels as well as in the inhibition of autophagy, suggesting that acetate may indeed represent the trigger of its downstream metabolic pathway (Figure S4C). To test if the increased protein level of Acs2p also resulted in higher enzyme activity, we determined the AcCoA synthetase (ACS) activity from crude protein extracts using an established biochemical enzyme assay (van den Berg et al., 1996), using Δ*acs1* background conditions to specifically address Acs2p activity (for details, see Supplemental Experimental Procedures and Figures S3J–S3L). In agreement with the idea that deletion of *ACH1* hyperactivates the nucleocytosolic AcCoA pathway, *ach1 acs1* double mutant cells developed an age-dependent increase in ACS activity compared to *acs1* control cells (Figure S4D).

Simple measurement of AcCoA levels is not applicable to our experimental question, as crude extracts reflect the total cellular content of AcCoA, not allowing for discrimination of the mitochondrial versus nuclear-cytosolic pools, which constitute two separate subcellular sites of AcCoA generation in yeast (Takahashi et al., 2006). Therefore, we decided to investigate the changes in AcCoA metabolism by alternative technologies that were based on the consideration that AcCoA represents an important cofactor of protein acetylation in eukaryotic cells and may directly affect the level of protein acetylation, enzymatically or nonenzymatically (Guan and Xiong, 2011). Consistent with the increased Acs2p activity, *ach1* mutants exhibited increased overall acetylation based on immunoblot analysis using pan-acetyl-lysine antibodies (Figure 4C, Input). In a more sophisticated approach using SILAC-based proteomics of crude protein extracts that were enriched for acetylated proteins by means of immunoprecipitation (IP) (Figure 4C, *Ac-Lys IP*), we identified potential targets affected by deletion of *ACH1*. Under the selected cutoff conditions (reproducible 1.5-fold regulation; for details, see Experimental Procedures), 61 proteins were found to be hyperacetylated upon *ACH1* deletion, while only 7 proteins were hypoacetylated (Figure 4D). Among these hyperacetylated target proteins, we identified several histones (Figure 4D, red highlighted text), in line with the prior observation that histone acetylation is associated with cellular AcCoA fluctuations in yeast and mammals (Cai et al., 2011; Wellen et al., 2009) as well as to the activity of Acs2p (Takahashi et al., 2006). The most abundant immunoblot signals detected after IP using pan-acetyl-lysine antibodies indeed corresponded to the size of histones at about 10–15 kDa (Figure 4C).

Next, we directly assessed the levels of histone H3 acetylation by means of acetylation-site-specific antibodies (i.e., histone marks that we have previously connected to autophagy during aging of yeast; Eisenberg et al., 2009). In line with the mass spectrometry results, Δ*ach1* but also Δ*mpc1* cells exhibited consistent hyperacetylation of all tested N-terminally located lysine residues in histone H3 compared to wild-type cells (Figures 4E, 4F, S4E, and S4F). As identified by our mass spectrometry analysis, the histones H2A and H2B appeared similarly hyperacetylated (Figure 4D, *HTA1/2 and HTB2*), as observed for H3. Since acetylation of histone H4, in particular that of lysine 16, was only recently found to regulate the outcome of autophagy in yeast and mammals (Füllgrabe et al., 2013) and may determine yeast replicative lifespan (Dang et al., 2009), we asked whether this histone was also affected by deletion of *ACH1*. Interestingly, histone H4 acetylation was unaffected by deletion of *ACH1* after 3 days of aging (Figure S4G), pointing toward a role of histone H2A/B and H3 acetylation under conditions of chronological aging that is distinct from that of the control of replicative lifespan by acetylation targets at histone H4.

In conclusion, impaired mitochondrial AcCoA production resulting from deletion of *ACH1* or *MPC1* causes upregulation of the nucleocytosolic pathway of AcCoA biosynthesis, as indicated by a concomitant increase in the Acs2p substrate acetate, the expression of Acs2p protein, the enzymatic activity of Acs2p, and hyperacetylation of specific histones.

### Episomal Overexpression of *ACS2* Mimics the Effects of *ACH1* Deletion

To exclude that the effects of *ACH1* or *MPC1* deletion were simply due to a general defect in mitochondrial AcCoA supply, we tested whether genetic overexpression of *ACS2* mimicked the effects of blocking mitochondrial AcCoA pathways. Indeed, Gal10p-driven overexpression of *ACS2* led to early onset cell death (Figures 4G and 4H) and limited the autophagic response during aging (Figures 4I and 4J). Importantly, ectopic expression of a mutant *acs2-Ts1* allele, which is stably expressed but shows reduced catalytic activity upon increased temperature (Takahashi et al., 2006), failed to induce cell death and caused reduced histone acetylation compared to expression of its wild-type counterpart that showed toxicity compared to BY4741 controls (Figures S4I–S4K). Of note, under our aging conditions (using only moderately increased permissive temperature to facilitate normal growth of the otherwise lethal *acs2-Ts* mutation), both the *ACS2* wild-type (Wt-ACS2) and the *Acs2-Ts1* mutant displayed a comparable increase in Acs2p levels compared to its BY4741 background strain (Figure S4H).

### Histone Hyperacetylation Is Associated with Impaired Transcription of Autophagy-Essential Genes

**Figure 5:**
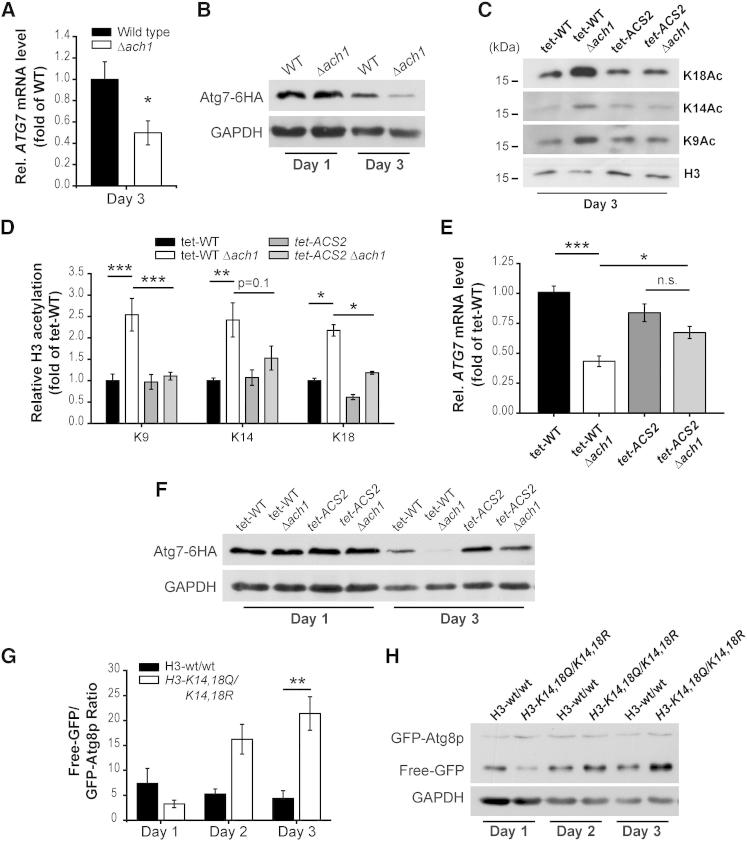
Acs2p Controls ATG7 Transcription through Epigenetic Histone Acetylation (A) *ATG7* mRNA levels by quantitative reverse-transcriptase PCR (RT-qPCR) of wild-type and Δ*ach1* cells aged to day 3 (see also Figure S5). Rel. mRNA levels are expressed as ratios of 18S rRNA normalized to wild-type cells by ΔΔCt-method. Data represent means ± SEM (n = 8). (B) Representative immunoblot analysis from wild-type (WT) and *ACH1*-deleted (Δ*ach1*) yeast expressing chromosomally tagged *ATG7* by C-terminal 6HA fusion aged to indicated time points. Blots were probed with anti-HA and anti-GAPDH (loading control) antibodies. (C and D) Representative immunoblots (C) and densitometric quantification (D) of whole-cell acid extracts of wild-type and Δ*ach1* cells combined with or without knockdown of *ACS2* (*tet-ACS2*). Cells were chronologically aged to day 3 in the presence of 1 ng/ml doxycycline. Blots were probed with antibodies against total histone H3 (loading control) or H3 acetylated lysines (K9Ac, K14Ac, K18Ac). Data represent means ± SEM (n = 8). (E and F) *ATG7* mRNA levels (E) by RT-qPCR and representative immunoblot analysis (F) of wild-type and Δ*ach1* cells with or without knockdown of *ACS2* (*tet-ACS2*) as in (C) and (E) aged to day 3. Rel. mRNA levels (E) are expressed as ratios to 18S rRNA normalized to wild-type cells by ΔΔCt method. Data represent means ± SEM (n = 7–8). (G and H) Representative immunoblot (H) and densitometric quantification (G) of histone H3 wild-type (H3-wt/wt) and H3 mutated (*H3-K14,18Q/-K14,18R*) strains carrying the GFP-Atg8p fusion to calculate “free-GFP/GFP-Atg8p Ratio” indicative of autophagic flux. Data represent means ± SEM (n = 4) (for strain details and supplemental data, see Figures S5D–S5F). ^∗^p < 0.05, ^∗∗^p < 0.01, and ^∗∗∗^p < 0.001; n.s., not significant.

We previously reported that histone hypoacetylation induced by supplementation of cultures with spermidine (an inhibitor of histone acetyl transferases) or simultaneous genetic inactivation of two histone acetyl transferase complexes correlates with enhanced expression of *ATG* genes and increased autophagy during aging (Eisenberg et al., 2009). Based on this premise, we hypothesized that histone hyperacetylation induced by upregulation of the Acs2p pathway could repress the transcription of autophagy-essential *ATG* genes. We therefore determined the relative mRNA abundance of five *ATG* transcripts (*ATG5*, *ATG7*, *ATG8*, *ATG11*, and *ATG14*) by quantitative reverse-transcriptase PCR. *ACH1* deletion significantly reduced the abundance of several *ATG* transcripts, including that of *ATG7*, whose mRNA level decreased to ∼40% compared to the wild-type level (Figures 5A and S5A). In contrast, the *ATG8* mRNA level increased in Δ*ach1* cells (Figure S5A). The protein levels of Atg7p (assessed by immunoblotting of *pATG7-ATG7-6HA* fusion strains) consistently decreased upon deletion of *ACH1* during aging (Figure 5B), while Atg8p levels remained constant (compare immunoblots of *pATG8-EGFP-ATG8* in Figure 2C). The level of total Atg7p also decreased significantly in aged wild-type cells (Figure 5B), again in clear contrast to Atg8p (Figure 2C). Thus, as exemplified by Atg7p, transcriptional control of the autophagy-relevant proteome may be crucial for enduring autophagic activity during chronological aging.

### *ACS2* Depletion Reinstalls Histone Deacetylation and *ATG7* Transcription in *ach1* Mutants

In order to test if Acs2p-induced histone acetylation was indeed causally linked to the transcriptional repression of *ATG7*, we tested if knockdown of *ACS2* in the background of *ACH1* deletion would (1) reverse the observed histone hyperacetylation phenotype and (2) reinstate normal *ATG7* transcription. Using the conditions depicted in Figure 1, knockdown of *ACS2* almost completely restored physiological (wild-type-like) levels of histone acetylation in *ach1* mutant cells (*tet-ACS2* Δ*ach1*), while deletion of *ACH1* alone (tet-WT Δ*ach1*) again increased overall acetylation at lysines 9, 14, and 18 of histone H3 compared to tet-WT controls (Figures 5C and 5D). Consistently, knockdown of *ACS2* also partly recovered *ATG7* mRNA and protein levels (Figures 5E and 5F, respectively) and almost completely annihilated the transcriptional defect induced by *ACH1* deletion (*tet-ACS2* Δ*ach1* compared to *tet-ACS2*; Figure 5E).

Altogether our data are consistent with a causal inverse relationship of histone H3 acetylation with the transcriptional control of autophagy-essential genes, exemplified by *ATG7* that is profoundly influenced by the Acs2p-mediated AcCoA production pathway. In support of a deficiency in the Atg7p-dependent Atg8p lipidating machinery, *ach1* mutants failed to accumulate lipidated Atg8p normally evident from enlarged GFP-Atg8p punctuate structures that were observed in autophagy-deficient *atg1* mutants carrying functional *ATG7* (Figure S5C; see also Autophagy Measurements in the Supplemental Experimental Procedures for more details).

In order to demonstrate the principal requirement of histone acetylation in age-associated autophagy control, we created a panel of (nonacetylable) histone H3 lysyl point mutations, mimicking different states of acetylation and deacetylation. In addition to pure deacetylation-mimicking lysine to arginine (KR) mutations (that likely result in quite unphysiological situations), we also rendered mixed KQ/KR mutations introduced to either one of two histone copies present within the genome as a promising strategy to test for more physiological alterations that retain a certain opening of the chromatin. The triple K9,14,18R mutation indeed resulted in generally impaired survival during aging (for summary of results and histone mutants tested, see Figure S5D), but strikingly we identified a mutant (H3-K14,18Q/K14,18R) that continuously enhanced autophagy during aging (Figures 5G, 5H, and S5D–S5F). Although such mutants are per se far away from the highly refined, time- and location-dependent chromatin modifications that occur in vivo, this finding demonstrates that epigenetic modifications by histone acetylation are in principle capable of modulating the cellular autophagic response during aging, and goes in line with our hypothesis that histone acetylation may represent a determining downstream event upon modulation of AcCoA availability.

### Depletion of *ACS2*, but Not Inhibition of Tor or Sch9 Signaling, Restores Autophagy in *ach1* Mutants

**Figure 6:**
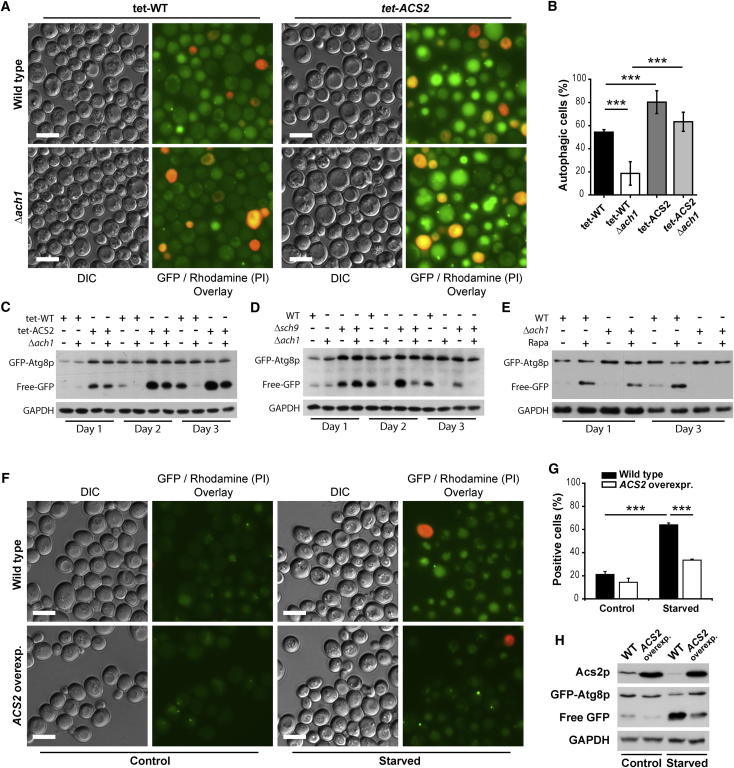
Knockdown of Acs2p Cures the Autophagy Defect in *ach1* Mutants (A and B) Representative micrographs (A) and respective quantification (B) of wild-type and Δ*ach1* cells expressing GFP-Atg8p chimera combined with or without knockdown of *ACS2* (*tet-ACS2*). Cells were chronologically aged to day 3 in the presence of 1 ng/ml doxycycline and PI counterstained prior to epifluorescence microscopy (see also Figure S6). (C) Representative immunoblot analysis of cells shown in (A) aged until days 1, 2, and 3 to detect “free-GFP” indicative of autophagic flux (see Figure S6A for quantification). (D and E) Representative immunoblot analyses of GFP-Atg8p expressing wild-type (WT) and Δ*ach1* cells either combined with deletion of *SCH9* (D) or supplemented with or without 20 nM rapamycin (Rapa) (E) and aged until indicated time points. Blots were probed with anti-GFP and anti-GAPDH (loading control) antibodies to detect “free-GFP” indicative of autophagic flux (see Figures S6B and S6C for quantification). (F and G) Representative micrographs (F) and respective quantification (G) of GFP-Atg8p expressing yeast cells ectopically overexpressing *ACS2 (ACS2 overexp.)* or carrying the empty vector (wild-type) during nutrient depletion (starved) compared to SC 2% galactose control conditions (control). Counterstaining of cells with propidium iodide was used to visualize dead cells. (H) Representative immunoblot analyses of the experiment shown in (F) to detect “free-GFP” indicative of autophagic flux. ^∗∗∗^p < 0.001.

As knockdown of *ACS2* in the background of Δ*ach1* almost completely abolished the transcriptional impairment of *ATG7*, we next asked whether *ACS2* knockdown would also be able to reinstate the autophagic activity of *ach1* mutant cells. Strikingly, knockdown of *ACS2* almost completely restored age-associated autophagy of *ach1* mutants (Figures 6A–6C and S6A), as determined by the frequency of cells with GFP-Atg8p-positive vacuoles (Figures 6A and 6B) and the levels of *liberated* GFP reflecting autophagic flux (Figures 6C and S6A). Autophagic activity of *tet-ACS2* Δ*ach1* cells approached that of the enhanced levels of the corresponding control strain without deletion of *ACH1* (*tet-ACS2*). Importantly, the knockdown of *ACS2* did not prevent extracellular accumulation of acetate induced by deletion of *ACH1*. The single deletion mutant (tet-WT Δ*ach1*), as well as *ACH1* deletion combined with the knockdown of *ACS2* (*tet-ACS2* Δ*ach1*), displayed almost comparable levels of excess acetate released from cells (Figure S6D). In addition, the autophagy-inhibitory effect of acetate supplementation to aging yeast cultures (compare Figure S4C) was annihilated and autophagic activity almost retained at wild-type levels in the *ACS2* knockdown condition (Figure S6E). This excludes a possible toxic effect of extracellular acetate on general cellular functions including autophagy and strongly argues for a specific role of the nucleocytosolic (Acs2p-mediated) pathway of acetate utilization that explains the inhibition of autophagy by *ACH1* deletion.

We next investigated whether the autophagy defect of cells lacking *ACH1* would be bypassed by inhibition of autophagy-regulatory kinases. Suppression of Tor- or Sch9-function by either genetic or pharmacological means induces autophagy and delays aging in yeast and other model organisms (He and Klionsky, 2009; Madeo et al., 2010a). Using previously reported conditions (Alvers et al., 2009), rapamycin, a potent inhibitor of TORC1, was able to induce autophagy during aging of BY4742 wild-type cells but completely failed to restore the age-induced autophagy defect of Δ*ach1* cells (Figures 6E and S6C). Rapamycin was well capable of inducing autophagy in young (day 1) Δ*ach1* cells (Figures 6E and S6C), excluding that rapamycin would simply fail to inhibit Tor in *ach1* mutant cells at the concentrations used for wild-type cells. Similarly, deletion of *SCH9*, which has been suggested to act in parallel to TORC1 as an autophagy suppressor (Yorimitsu et al., 2007) as well as in the same pathway with TORC1 during lifespan regulation (Wei et al., 2009), transiently amplified the autophagic response of aging wild-type but again failed to reinduce autophagy of aging *ach1* cells (Figures 6D and S6B). Thus, knockdown of *ACS2*—but not inhibition of Tor or Sch9—specifically reinstated autophagy of *ach1* mutants, strengthening our conclusion that upregulation of the Acs2p pathway is causally linked to the autophagy defect of Δ*ach1* cells.

Finally, we tested if ectopic overexpression of *ACS2* would also impair autophagy induced by nutrient depletion, a known autophagy trigger via nutrient-responsive signaling, including TORC1- or AMPK-dependent pathways (He and Klionsky, 2009). While the level of Acs2p decreased upon starvation (Figure 6H, compare empty vector controls, “*WT*”), *ACS2* overexpression profoundly inhibited autophagic flux after 12 hr of starvation, as evident from reduced vacuolar translocation of GFP-Atg8p (Figures 6F and 6G) as well as reduced amount of *liberated* GFP (Figure 6H, “Free-GFP”). Similar results were observed after autophagy induction by rapamycin treatment (data not shown).

In sum, enduring autophagic activity during aging (but also that of acute nutrient depletion conditions) is profoundly repressed by nucleocytosolic Acs2p, which appears dominant over autophagy regulation by known kinase regulators such as Sch9 or TORC1.

### Reinstating Autophagy of Δ*ach1* Partly Restores Lifespan in an Autophagy-Dependent Manner

**Figure 7:**
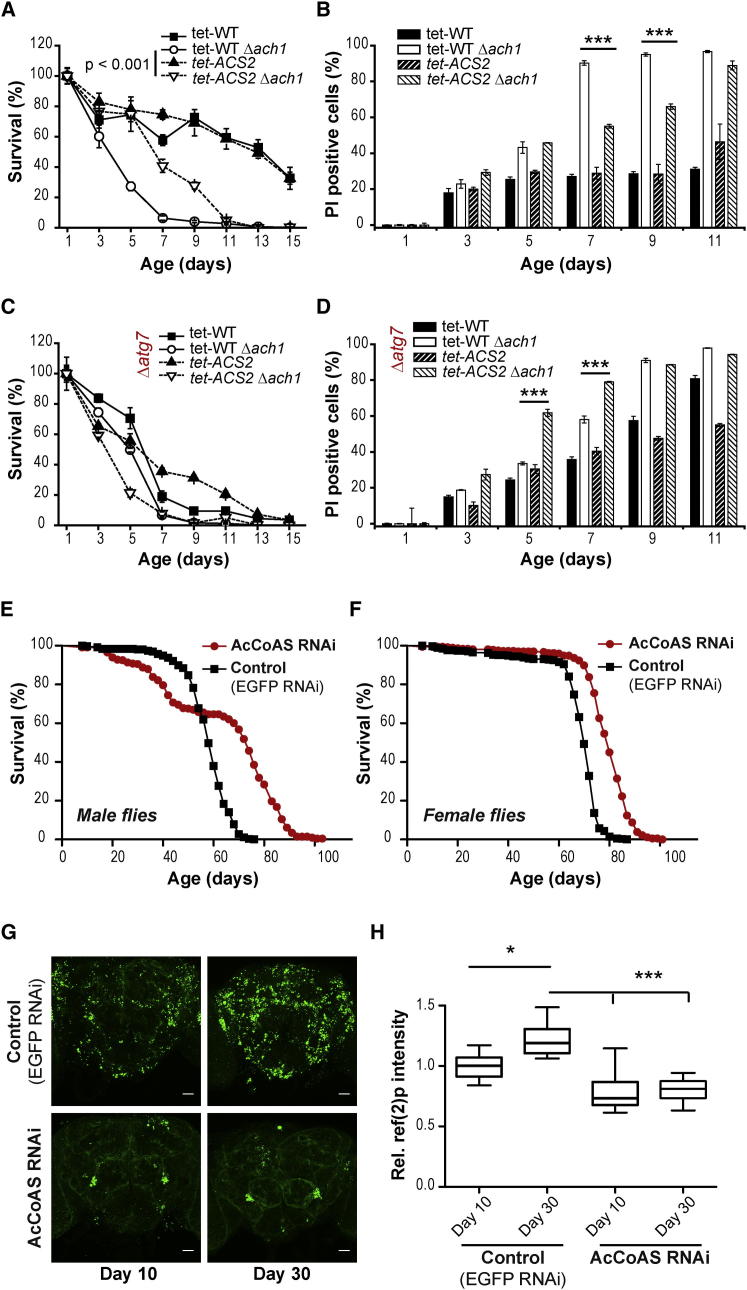
Knockdown of Acs2p Partly Restores Survival of *ach1* in an *ATG7*-Dependent Manner (A and B) Chronological aging in SC 2% glucose medium supplemented with 1 ng/ml doxycycline of wild-type and Δ*ach1* cells combined with or without knockdown of *ACS2* (*tet-ACS2*). Survival (A) was determined by colony-forming capacity (clonogenicity). Age-associated cell death (B) was assessed through propidium iodide (PI) staining analyzed by flow cytometry. Data represent day 1 normalized means ± SEM (n = 4). (C and D) Survival (C) and cell death (D) of chronological aging experiment similar to (A) and (B) but in the background of *ATG7*-deleted autophagy-incompetent cells (Δ*atg7*). Data represent day 1 normalized means ± SEM (n = 4). (E and F) *Drosophila* lifespan analyses of male (E) and female (F) flies depleted for acetyl-CoA synthetase (AcCoAS) using RNAi-mediated knockdown (*AcCoAS RNAi*) compared to isogenized controls (*Control (EGFP RNAi)*). Log rank tests revealed p < 0.0001 for both sexes. (G) Immunofluorescence specific to p62-homolog ref(2)p of adult brain sections from 10-day-old flies compared to 30-day-old flies as depicted in (E). Representative confocal micrographs are shown with scale bars representing 25 μm. (H) Quantification of total ref(2)P intensity in the central brain region normalized to 10-day-old control flies (*Control (EGFP RNAi)*). Standard box plot represents data from seven to eight independent brains (whiskers indicate minimum and maximum values). ^∗^p < 0.05, ^∗∗∗^p < 0.001.

Given the crucial role of autophagy for healthy aging, reinstating autophagy in *ach1* mutants may recover survival of aging cells, which would hence argue in favor of a causal role of the autophagy deficit in the reduction of lifespan. Knockdown of *ACS2*, using the same conditions that reactivated autophagy of *ach1* cells, indeed significantly enhanced survival (Figure 7A) and reduced markers of cell death (Figure 7B) in aging *ach1* cells. Compared to that of wild-type cells, the strong lifespan-shortening effect of *ACH1* deletion (tet-WT versus tet-WT Δ*ach1*) was significantly inhibited by knockdown of *ACS2* (*tet-ACS2* versus *tet-ACS2* Δ*ach1*). Additional deletion of the autophagy-essential gene *ATG7* (known to completely abrogate autophagy in aging yeast) again revealed the full lifespan-compromising consequences of *ACH1* deletion, even when *ACS2* expression was suppressed (Figures 7C and 7D). Knockdown of *ACS2* even accelerated death of *ach1* mutants when autophagy was absent (compare *tet-ACS2* Δ*ach1* Δ*atg7* to tet-WT Δ*ach1* Δ*atg7*, Figures 7C and 7D). This strongly suggests that loss of autophagy is—at least in part—responsible for the detrimental effects of *ACH1* deletion.

### Knockdown of Acetyl-CoA Synthetase Extends Mean and Maximum Lifespan of Flies

Finally, we tested if inhibition of the nucleocytosolic acetyl-CoA production pathway would affect autophagy and aging in a metazoan organism. In *Drosophila*, a renowned model of aging in a higher eukaryote, autophagic efficacy was shown to decline with brain aging, while activating the autophagic pathway by brain-specific overexpression of Atg8a using brain-specific appl-gal4 was previously shown to improve lifespan (Simonsen et al., 2008). Therefore, we addressed the effects of brain-specific RNAi-mediated knockdown of the fly acetyl-CoA synthetase gene (AcCoAS).

Remarkably, although initially with adverse effects in males, flies expressing RNAi specific for AcCoAS exhibited mean and maximum lifespan extension in both sexes compared to isogenic control flies that expressed an unrelated RNAi specific for GFP not present in this organism (Figures 7E and 7F, p < 0.0001 for both sexes). We then assessed the levels of the p62-homolog ref(2)p, a protein known to increase with age and to decrease again when autophagy is activated (Gupta et al., 2013). In fact, while both the number of dot-like (aggregated) ref(2)p structures as determined by immunofluorescence (Figure 7G) and overall levels of ref(2)p as quantified by total pixel intensities (Figure 7H) increased in brains of aged (30 days) compared to young (10 days) control animals, this increase was largely abolished in the animals expressing the AcCoAS RNAi by appl-gal4. Thus, our data suggest that, indeed, knockdown of AcCoAS induced the autophagic pathway associated with its longevity-promoting effects.

## Discussion

In the present study we identified the nucleocytosolic (Acs2p-mediated) AcCoA production pathway as a suppressor of age-associated autophagy, conserved from yeast to flies. While upregulation of Acs2p activity as a consequence of impaired mitochondrial acetate or pyruvate utilization led to autophagy deficiency, reducing the protein level of Acs2p was sufficient to ameliorate the autophagic response to aging (see Figure S7 for an overview of the mechanistic model). In agreement with this idea, hyperactivation of the Acs2p pathway observed in *ach1* or *mpc1* mutant cells hampered genetic or pharmacological means of autophagy induction, including deletion of *SCH9* or application of rapamycin, respectively. Ectopic overexpression of *ACS2* not only limited the autophagic response to aging but also strongly impaired autophagy induced by nutrient depletion, a scenario known to inhibit nutrient signaling. Thus, autophagy observed under distinct conditions is counteracted by a hyperactive Acs2p pathway, which may imply a general requirement of Acs2p inhibition for efficient induction of autophagy.

The activity of Acs2p culminates in its downstream target of histone acetylation. The mechanism by which Acs2p activity affects age-associated autophagy may thus include epigenetic control of *ATG* gene transcription, as exemplified in this study by regulation of *ATG7* mRNA and protein levels. *ATG7* mRNA and protein levels inversely correlated with the acetylation of N-terminally located lysyl residues of histone H3. However, regulation of autophagy by changes in the relevant transcriptome most likely involves more than just one *ATG* transcript. The precise mechanism of how global hyperacetylation modulates the autophagy-relevant transcriptome remains to be clarified but is in agreement with our previous finding that global hypoacetylation can induce *ATG7* transcription and autophagy upon treatment with the natural polyamine spermidine (Eisenberg et al., 2009; Madeo et al., 2010b). Our findings add evidence to a previously underestimated pathway of autophagy regulation that may particularly apply to enduring autophagic responses and that involves epigenetic and/or transcriptional alterations (Füllgrabe et al., 2014). Acetylation of histone H4 at lysine 16 has only recently been shown to direct the decision of whether or not autophagy can become toxic (Füllgrabe et al., 2013). The transcription factor FoxO3 controls the expression of autophagy-related genes, including that of LC3—the mammalian ortholog of yeast *ATG8*—and is required for autophagy induction of mammalian muscle cells with implications to muscle atrophy (Zhao et al., 2007). In yeast, *ATG8* transcription is regulated by the Ume3-Sin3-Rpd3-complex, which suppresses induction of *ATG8* under nonstarved conditions and modulates the size of autophagosomes during starvation-induced autophagy (Bartholomew et al., 2012).

In this line and as a proof of principle we introduced a histone point mutation that displayed enhanced autophagy during aging. However, we cannot exclude that in combination with transcriptomic alterations driven by epigenetic histone modifications also acetylation of nonhistone proteins may explain the observed phenotypes. This could, for instance, include the acetylation of Atg proteins or autophagy-relevant transcription factors that affect *ATG* gene transcription more directly. Apart from protein acetylation and/or histone acetylation, more indirect effects on metabolism downstream of AcCoA generation may also explain some of the consequences on autophagy and lifespan and need to be investigated in the future.

The connection of metabolism to posttranslational modifications of histones has been recognized and linked to disease-relevant situations (Kaelin and McKnight, 2013; Lu and Thompson, 2012). However, the connection of AcCoA metabolism to age-relevant autophagy has remained elusive. Here, we describe how energy metabolism controls autophagic activity during cellular aging through modulation of a central metabolite, AcCoA, presumably via its connection to epigenetic changes in chromatin.

The role of acetate in promoting yeast aging has been critically discussed recently (Burhans and Weinberger, 2009; Burtner et al., 2009; Longo et al., 2012). Our data strongly suggest that acetate exerts its proaging effects on yeast not only through rather unspecific events resulting from the primary toxic activity of extracellular acetate, but also through the Acs2p-mediated conversion of acetate to AcCoA. Importantly, acetate metabolism was proposed to contribute to aging in mammals, again via its impact on protein acetylation by generation of AcCoA and subsequent regulation of metabolism (Shimazu et al., 2010).

Interestingly, many lifespan-prolonging regimens (e.g., caloric restriction, rapamycin treatment) or age-associated disease-preventing supplements (e.g., resveratrol) that are known to induce autophagy were also reported to modulate mitochondrial function and respiration (Bonawitz et al., 2007; Houtkooper et al., 2010; Ocampo et al., 2012; Pan et al., 2011). Hence, it is tempting to speculate that efficient utilization of AcCoA by mitochondrial TCA cycle activity and oxidative phosphorylation prevents overflow of AcCoA into protein acetylation and/or histone acetylation pathways that would negatively influence autophagy and other prosurvival processes during aging and age-associated disease.

In summary, we propose that the nucleocytosolic acetyl-CoA pool functions as a crucial, evolutionary conserved, and dominant inhibitor of autophagy and healthy aging. Inhibition of major nutrient signaling pathways (i.e., TORC1 or Sch9) fails to induce autophagy when the Acs2p pathway is activated, arguing for a fundamental function of this pathway downstream of nutrient signaling. Since AcCoA integrates various nutrition pathways, our findings have implications for the understanding of lifespan and autophagy regulation in the context of excessive, restricted, or unbalanced feeding behaviors.

## Experimental Procedures

### Yeast Strains, Molecular Biology, and Chronological Aging Experiments

Experiments were carried out in *S. cerevisiae* BY4741 or BY4742 wild-type yeast and respective mutant strains depicted in Table S1. Chronological aging experiments were performed in SC 2% glucose medium, survival determined by plating cells on YPD agar (clonogenicity), and cell death quantified by propidium iodide staining. For further details on yeast strains and genetics (Table S1), pharmacological treatments, cell death assays, and culture conditions, please refer to Supplemental Information.

### Immunoblotting and Quantification of Histone Acetylation

Immunoblotting was performed using standard protocols. Details on antibodies and on quantification of histone acetylation by blotting serial dilutions of acid extracts are depicted in Supplemental Information.

### Yeast Autophagy Measurements

Autophagy was measured either by monitoring the cytosol to vacuole translocation of Atg8p using fluorescence microscopy or immunoblotting (*GFP liberation assay*) of cells/cell extracts from strains carrying a GFP-Atg8p fusion protein (Kirisako et al., 1999; Klionsky et al., 2007) expressed from its endogenous promoter and natural chromosomal locus (*pATG8-EGFP-ATG8* strains) or by alkaline phosphatase (ALP) activity (Noda and Klionsky, 2008). Propidium iodide (PI) staining served to exclude analyses of dead, potentially autofluorescent cells visualized by standard rhodamine filters. For more details also on quantification of results, refer to Supplemental Information.

### Determination of Acetyl-Proteome from SILAC Yeast Cultures

To assess changes in the acetyl-proteome, yeast protein extracts were first enriched for acetylated proteins by IP. Stable isotope-labeled amino acid cell cultures (SILAC) from yeast (Lys0, Lys4, or Lys8 labeled) were subjected to cell disruption by glass beads in the presence of histone deacetylase (trichostatin A, 30 mM) and sirtuin (nicotinamid, 2 μM) inhibitors. Crude extracts from wild-type and Δ*ach1* cultures aged to day 3 were mixed in equal amounts of total protein (determined by Bio-Rad protein assay, Bio-Rad) and subjected to IP using pan-acetyl-lysine antibodies (1:100, Cell Signaling; an equal mix of Ac-K [#9441] and Ac-K^2^ [#9814] antibodies was used) and subsequently analyzed by mass spectrometry. For details on IP procedure and on quantification and identification of SILAC ratios by mass spectrometry, see Supplemental Information.

### Acetyl-CoA-Synthetase Activity

Acetyl-CoA synthetase (ACS) activity was determined using an established biochemical enzyme assay (van den Berg et al., 1996). For details, see Supplemental Information.

### Acetic Acid Detection

Enzymatic measurement of acetic acid from crude culture supernatants (appropriately diluted with water) was conducted using the Acetic Acid (Acetate Kinase Manual Format) kit (Megazyme) following the manufacturer’s protocol adapted to a volume of 100 μl for readout in a TECAN plate reader.

### Quantitative Reverse-Transcriptase PCR

Target mRNA quantification by quantitative reverse-transcriptase PCR using ΔΔCt-method with 18S rRNA as an internal standard was performed on an ABI StepOnePlus using SYBR Select Master Mix (Life Tech, Invitrogen) ΔΔCt-method. Primers (Table S3) and details can be found in Supplemental Experimental Procedures.

### *Drosophila* Lifespan Analyses and Brain Immunofluorescence

UAS-RNAi lines for acetyl-CoA Synthetase (P{TRiP.HMS02314}attP2) and EGFP (P{VALIUM20-EGFP.shRNA.1}attP2) serving as a background-matched control were obtained from Bloomington *Drosophila* Stock Center. Isogenized panneural driver line APPL-Gal4 was used to drive RNAi expression in fly brains. Details on housing, survival assessment, and ref(2)p-specific immunofluorescence can be found in Supplemental Information.

### Statistical Analyses

One-factor analysis of variance (ANOVA) corrected by the post-hoc Bonferroni test was used for all experiments (multiple comparisons as appropriate) except for mass spectrometric and for chronological lifespan (CLS) analyses. If not otherwise stated, representative CLS experiments are shown with three to four biologically independent samples (as indicated) aged at the same time. CLS experiments have been performed at least three times with similar outcome. For comparison of CLS, a two-factor ANOVA with time and strain as independent factors was used. Log rank tests were performed for *Drosophila* lifespan analyses. Mass spectrometry was performed twice from two independent chronological aging experiments. Error bars represent SEM of biological replicates as indicated. Whiskers in box plots (Figure 7H) indicate minimum and maximum values.

## Supplemental Information

Document S1. Figures S1–S7, Tables S1–S4, and Supplemental Experimental Procedures
