# Supplementary material for: Nucleocytosolic Depletion of the Energy Metabolite Acetyl-Coenzyme A Stimulates Autophagy and Prolongs Lifespan
Source: Cell Metab. 2014 Mar 4;19(3):431–44. doi: 10.1016/j.cmet.2014.02.010 (PMC3988959; doi:10.1016/j.cmet.2014.02.010)
Supplement: Document S1. Figures S1–S7, Tables S1–S4, and Supplemental Experimental Procedures [file mmc1.pdf]

Cell Metabolism, Volume *19*

## Supplemental Information

# Nucleocytosolic Depletion of the Energy Metabolite Acetyl-Coenzyme A Stimulates Autophagy and Prolongs Lifespan

Tobias Eisenberg, Sabrina Schroeder, Aleksandra Andryushkova, Tobias Pendl, Victoria Küttner, Anuradha Bhukel, Guillermo Mariño, Federico Pietrocola, Alexandra Harger, Andreas Zimmermann, Tarek Moustafa, Adrian Sprenger, Evelyne Jany, Sabrina Büttner, Didac Carmona-Gutierrez, Christoph Ruckenstuhl, Julia Ring, Wieland Reichelt, Katharina Schimmel, Tina Leeb, Claudia Moser, Stefanie Schatz, Lars-Peter Kamolz, Christoph Magnes, Frank Sinner, Simon Sedej, Kai-Uwe Fröhlich, Gabor Juhasz, Thomas R. Pieber, Jörn Dengjel, Stephan J. Sigrist, Guido Kroemer, and Frank Madeo

## Supplemental Information to

### „Nucleo-cytosolic depletion of the energy metabolite acetyl-coenzyme A stimulates autophagy and prolongs life span"

**Inventory of Supplemental Information**

Supplemental Information includes seven figures, four tables and Supplemental Experimental Procedures providing additional details to yeast genetics and culture conditions, autophagy measurements, immunoprecipitation of acetylated proteins and mass spectrometric analyses, ACS activity assay, quantitative reverse-transcriptase PCR as well as Drosophila experiments.

## Supplemental Data

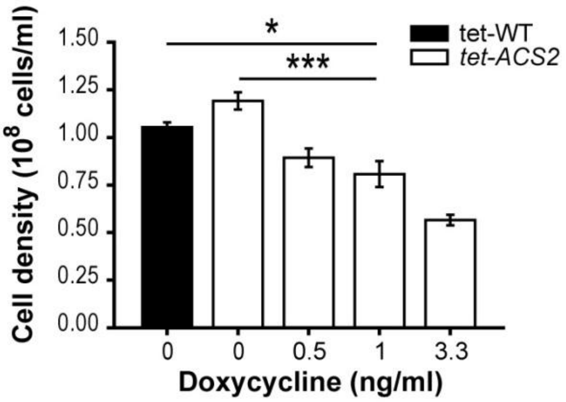


**Figure S1, related to Figure 1**

***Moderate reduction of cell densities indicates functional knockdown of ACS2***

Cell densities (cells/ml) were determined using CASY cell counter technology (Schaerfe System, Roche). Wild type cells (tet-WT) and cells carrying a doxycycline-repressible tet-O7 promoter controlling *ACS2* transcription expressing GFP-Atg8p chimera (see also Figure 1) were grown for 24 hours in the presence or absence of indicated doxycycline concentrations supplemented to SC 2% glucose medium. 1 ng/ml doxycycline showing significant functional reduction of *ACS2* evident from mild reduction of cell densities compared to tet-WT cells was selected to facilitate knockdown of *ACS2* throughout the study. Data represent means ± SEM (n = 4). *p < 0.05 and ***p < 0.001

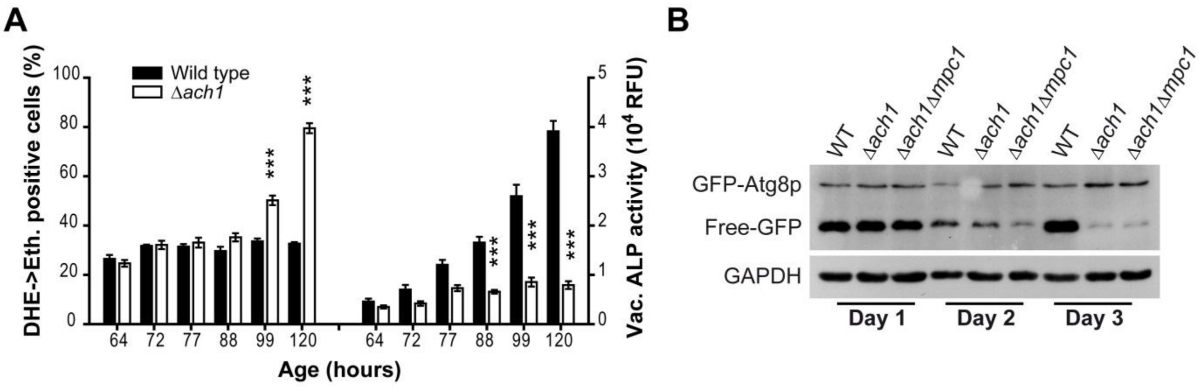


**Figure S2, related to Figure 2**

***Age-induced autophagy-impairment of ach1 precedes cell stress and death***

(**A**) Vacuolar alkaline phosphatase (Vac. ALP) activity indicative of autophagic flux was determined along with dihydroethidium to ethidium conversion (DHE->Eth.) as a measure of cell stress and death analyzed by flow cytometry. Strains carrying genetically engineered form of Pho8p lacking its N-terminal transmembrane domain (Pho8pΔN60) were chronologically aged to indicated time points and subjected to ALP activity assay with 1 µg total protein. Data represent means ± SEM (n = 8-12). ***p < 0.001

(**B**) Representative immunoblot analyses of wild type (WT), Δ*ach1* and Δ*ach1*Δ*mpc1* double-mutant cells. All strains were expressing GFP-Atg8p chimera and aged until indicated time points. Blots were probed with anti-GFP and anti-GAPDH (loading control) antibodies to detect 'Free-GFP' indicative of autophagic flux.

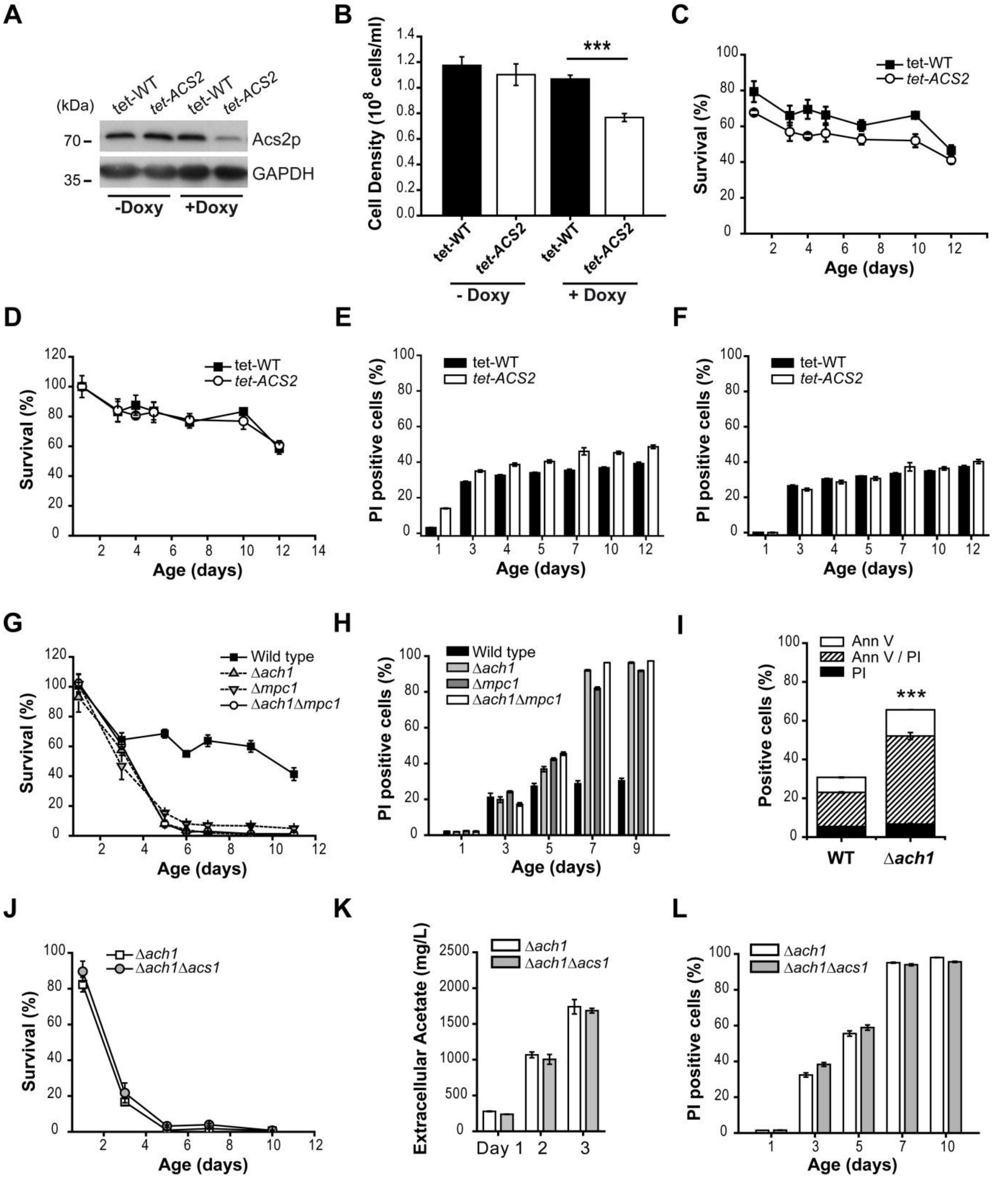


**Figure S3, related to Figure 3**

***Chronological life span remains unaffected by moderate knockdown of ACS2***

**(A)** Representative immunoblot using Acs2p- and GAPDH-specific (loading control) antibodies of protein extracts from wild type (tet-WT) and *ACS2* knockdown yeast (*tet-ACS2*). Cells were grown in SC 2% glucose medium for 24 hours (day 1 of aging) in the presence (+Doxy) or absence (-Doxy) of 1 ng/ml doxycycline to facilitate knockdown of *ACS2* by the doxycycline-repressible tet-promoter (see also Figure 1 and Figure S1).

**(B)** Cell densities (cells/ml) determined using CASY cell counter technology of cells depicted in (A) demonstrating moderate functional knockdown of Acs2p. Data represent means ± SEM (n = 4). ***p < 0.001

**(C-F)** Chronological life span (CLS) analyses in SC 2% glucose medium of wild type (tet-WT) compared to Acs2p depleted cells (*tet-ACS2*) as described in (A). Survival (C, D) was determined by clonogenicity and cell death (E, F) assessed by propidium iodide (PI) positive cells analyzed by flow cytometry. Data represent means ± SEM (n = 4) of a representative aging experiment, depicted as absolut (C, E) or day 1 normalized values (F, G).

(**G, H**) Chronological life span (CLS) analyses in SC 2% glucose medium of wild type (WT) Δ*ach1*, Δmpc*1* or Δ*ach1*Δmpc*1* double mutant cells. Survival (G) was determined by colony forming capacity (clonogenicity) and cell death (H) assessed by propidium iodide (PI) positive cells analyzed by flow cytometry. Data represent means ± SEM (n = 4) of a representative aging experiment.

(**I**) Quantification (flow cytometric analysis) of phosphatidylserine externalization and loss of membrane integrity using Annexin V (Ann V)/ propidium iodide (PI) costaining (at day 5) of chronologically aged wild type and Δ*ach1* cells (see Figure 3C). Consistent with earlier reports (Orlandi et al., 2012) and with the knowledge that cellular demise during aging of yeast is associated to programmed cell death processes (Fabrizio et al., 2005; Herker et al., 2004), *ach1* mutant cells exhibited increased fractions of early apoptotic ($AnnV^+/PI^-$) and secondary necrotic ($AnnV^+/PI^+$) cells. Data represent means ± SEM (n = 4). ***p < 0.001

(**J-L**) Chronological life span (CLS) analyses in SC 2% glucose medium of Δ*ach1* or Δ*ach1*Δ*acs1* double mutant cells performed in parallel to the experiment shown in Figure 3A. Survival (J) was determined by colony forming capacity (clonogenicity) and cell death (L) assessed by propidium iodide (PI) positive cells analyzed by flow cytometry. Extracellular acetate (K) was assessed from crude culture supernatants obtained at indicated time points of the CLS experiments shown in (J). Data represent means ± SEM (n = 4) of a representative aging experiment.

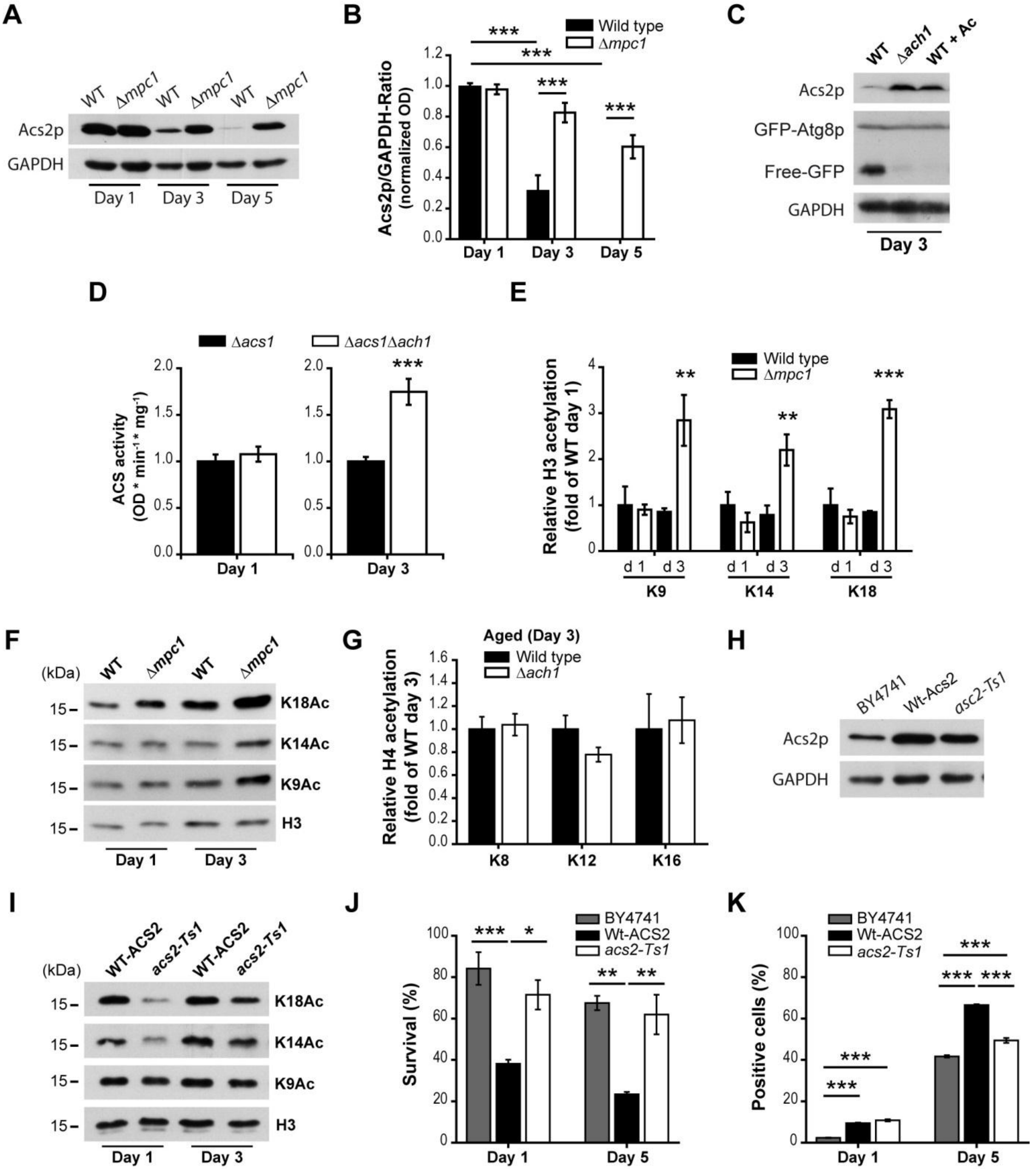


**Figure S4, related to Figure 4**

***Deletion of ACH1 or MPC1 leads to upregulation of nucleo-cytosolic Acs2p-dependent pathway crucial for histone H3 acetylation.***

**(A, B)** Representative immunoblot (A) and densitometric quantification expressed as normalized Acs2p/GAPDH-ratios (B) of protein extracts from wild type (WT) and *MPC1* deleted (Δ*mpc1*) yeast chronologically aged to indicated time points in SC 2% glucose medium. Blots were probed with anti-ACS2p and anti-GAPDH (loading control) antibodies.

**(C)** Representative immunoblot of protein extracts from three day old wild type cells expressing GFP-ATG8 with and without supplementation of 20 mM acetate (30 hours after inoculation) compared to Δ*ach1* cells of the same age. Blots were probed with anti-Acs2p and

anti-GAPDH (loading control) antibodies as well as anti-GFP antibody to assess '*Free-GFP*' indicative of autophagic flux.

**(D)** Relative Acetyl-CoA synthetase activity (Rel. ACS activity) of 'wild-type' *ACH1* containing (Δ*acs1*) and *ACH1*-deleted (Δ*acs1* Δach1) cells in the background of Δ*acs1* yeast strains and thus indicative of Acs2p-specific activity. Cells were aged to day 1 or day 3 and crude protein extracts subjected to ACS activity assay generating NADH assessed by absorbance at 340 nm (OD). NADH production per min and µg protein was normalized to 'wild-type' (Δacs1) conditions. Data represent means ± SEM (n = 32)

**(E, F)** Representative immunoblots of whole cell acid extracts of wild type (WT) and Δ*mpc1* cells chronologically aged to designated time points. (E) Blots were probed with antibodies against total histone H3 (loading control) or H3 acetylation sites (Ac-K) at the indicated lysine residues (K9, K14, K18). Densitometric quantification (F) of relative acetylation was calculated as Ac-K/total H3 ratios normalized to ratios of WT at day 1. Data represent means ± SEM (n = 7-8).

**(G)** Densitometric quantification of immunoblots obtained from extracts of aged (3 day old) Wild type and *ach1* mutant cells (not shown) probed with antibodies against total histone H4 (loading control) or H4 acetylation sites (Ac-K) at the indicated lysine residues (K8, K12, K16). Relative acetylation was calculated as Ac-K/total H4 ratios normalized to ratios of WT at day 3. Data represent means ± SEM (n = 4).

**(H, I)** Representative immunoblot (expressed as normalized Acs2p/GAPDH-ratios of protein extracts from wild type BY4741 (BY4741), temperature-sensitive *acs2* mutant (*acs2-Ts1*) and its corresponding wild type strain (*WT-ACS2*). Blots were probed with anti-ACS2p and anti-GAPDH (loading control) antibodies (H) or with antibodies against total histone H3 (loading control) or H3 acetylation sites (I) at the indicated lysine residues (K9, K14, K18).

(**J, K**) Survival during aging in SC 2% glucose medium at 28 °C (slightly increased permissive temperature) of cells presented in (H). Survival (J) was determined by colony forming capacity (clonogenicity) and cell death (K) assessed by propidium iodide (PI) positive cells analyzed by flow cytometry at indicated time points. Data represent means ± SEM (n = 4) of a representative aging experiment.

*p < 0.05, **p < 0.01 and ***p < 0.001

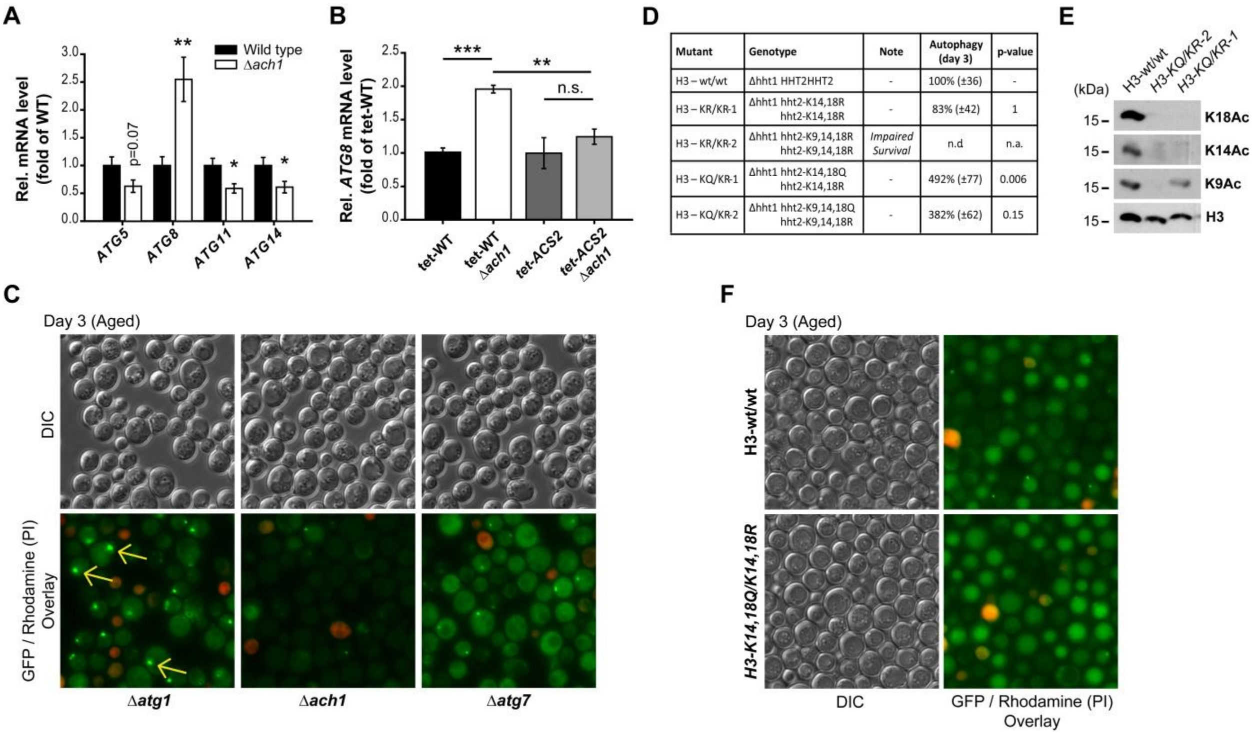


**Figure S5, related to Figure 5**

***Epigenetic chromatin modification by H3 acetylation affects autophagy-relevant transcription and determines autophagic flux during aging.***

**(A)** *ATG* mRNA levels (as indicated) by quantitative reverse-transcription real-time PCR (RT-qPCR) of wild type and Δ*ach1* cells aged to day 3 (see also Figure 5). Rel. mRNA levels are expressed as ratios of 18S rRNA normalized to wild type cells by ΔΔCt-method. Data represent means ± SEM (n = 6-8).

**(B)** *ATG7* mRNA levels by RT-qPCR of wild type and Δ*ach1* cells with or without knockdown of *ACS2* (*tet-ACS2*) aged to day 3 (compare to Figure 3C, D). Rel. mRNA levels are expressed as ratios of 18S rRNA normalized to wild type cells by ΔΔCt-method. Data represent means ± SEM (n = 7-8).

**(C)** Representative micrographs of pATG8-GFP-Atg8p expressing cells in the background of *ATG1*- (autophagy-deficient, but Atg8p-lipidation competent), *ACH1*- or *ATG7*- (autophagy-deficient due to lack of Atg8p-lipidation) deleted yeast strains. Yellow arrows indicate punctuate localized, presumably lipidated GFP-Atg8p.

**(D)** Table summarizing the results from a screen of four histone H3 point mutants carrying lysine to arginine (KR) or lysine to glutamine (KQ) at indicated histone sites, mimicking different states of (de)acetylation. Mutants were introduced to one of the two genes coding for identical histone H3 proteins (*HHT2*) in the background of *HHT1* deletion mutant. To facilitate wild type-like histone levels, H3 wild type strains (H3-wt/wt) as well as mutant strains (as indicated) carried two copies of *HHT2* gene. Autophagic activity was assessed by Free-GFP/GFP-Atg8p Ratio in cells expressing GFP-ATG8 from its endogenous promoter. Autophagy was not determined (n.d.) in strains showing considerable survival deficits during

aging. Data represent means ± SEM (n = 4) normalized to wild type conditions (100% autophagic activity).

**(E)** Immunoblots of acid extracts from H3 wild type or H3 mutant strains using antibodies recognizing total histone H3 (loading control) or indicated acetylated lysine residues to control for respective mutant conditions.

**(F)** Representative micrographs of pATG8-GFP-Atg8p expressing cells of wild type histone H3 (H3-wt/wt) or mutant histone-H3 (as indicated) after three days of aging on SC 2% glucose medium to visualize vacuolar localization of GFP-Atg8 indicative of autophagy.

*p < 0.05, **p < 0.01 and ***p < 0.001; n.s. = not significant

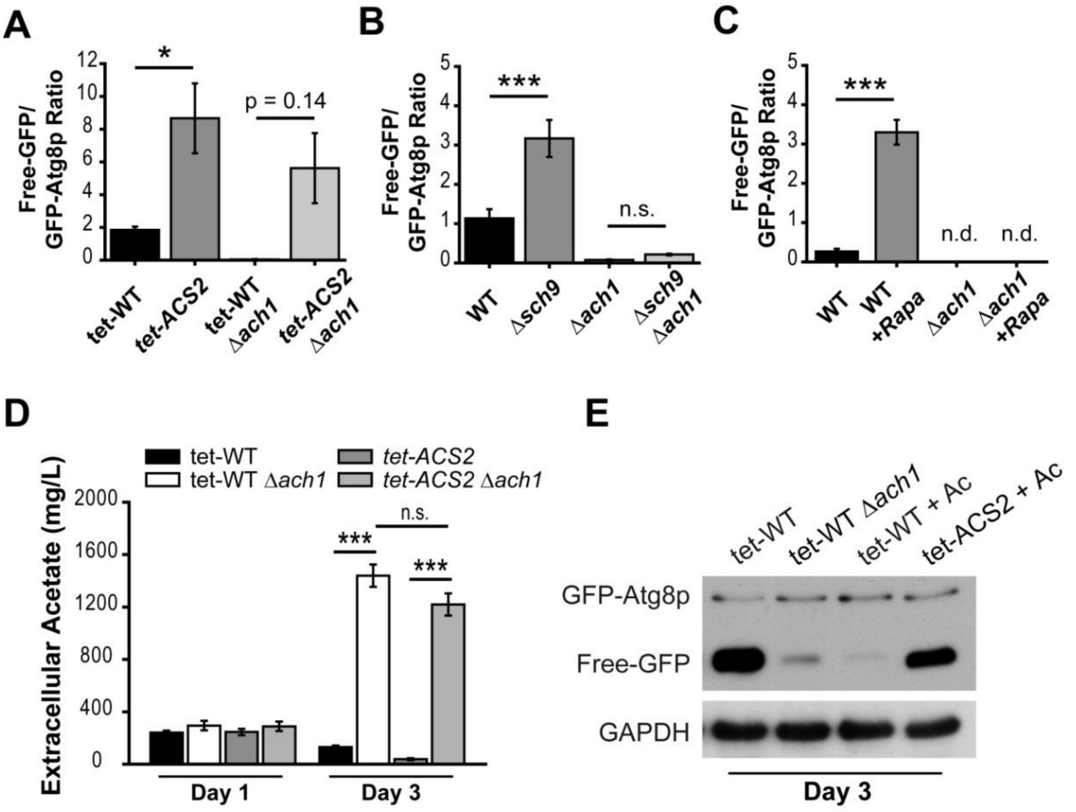


**Figure S6, related to Figure 6**

***Knockdown of ACS2 cures the autophagy defect of ach1 mutant cells, while excess acetate release remains unchanged.***

**(A-C)** Densitometric quantification (Free-GFP/GFP-Atg8p Ratio indicative of autophagic flux) of immunoblots shown in Figure 6C-E of wild type and Δ*ach1* cells expressing GFP-Atg8p chimera either combined with or without knockdown of *ACS2* (*tet-ACS2*) (A), combined with or without deletion of *SCH9* (B), or supplemented with or without 20 nM rapamycin (C). Strains were aged for two (B) three (A+C) days. Data represent means ± SEM (n = 4).

**(D)** Extracellular acetate assessed from crude culture supernatants obtained at indicated time points of wild type and Δ*ach1* cells with or without knockdown of *ACS2* (*tet-ACS2*). Data

were pooled from both cells expressing GFP-Atg8p chimera as well as respective background (wild-type ATG8) cells (compare Figures 5 and 6). Data represent means ± SEM (n = 12).

**(E)** Representative immunoblot analyses of wild type (tet-WT) and ACS2 knockdown (tet-ACS2) cells aged in the presence of 1 ng/ml doxycycline to day 3 with or without the addition of acetate compared to sole deletion of ACH1. All strains were expressing GFP-Atg8p chimera and blots were probed with anti-GFP and anti-GAPDH (loading control) antibodies to detect 'Free-GFP' indicative of autophagic flux. Acetate (20 mM) was supplemented as indicated to aging cultures 40 hours after inoculation.

*p < 0.05 and ***p < 0.001; n.s. = not significant; n.d. = not detectable

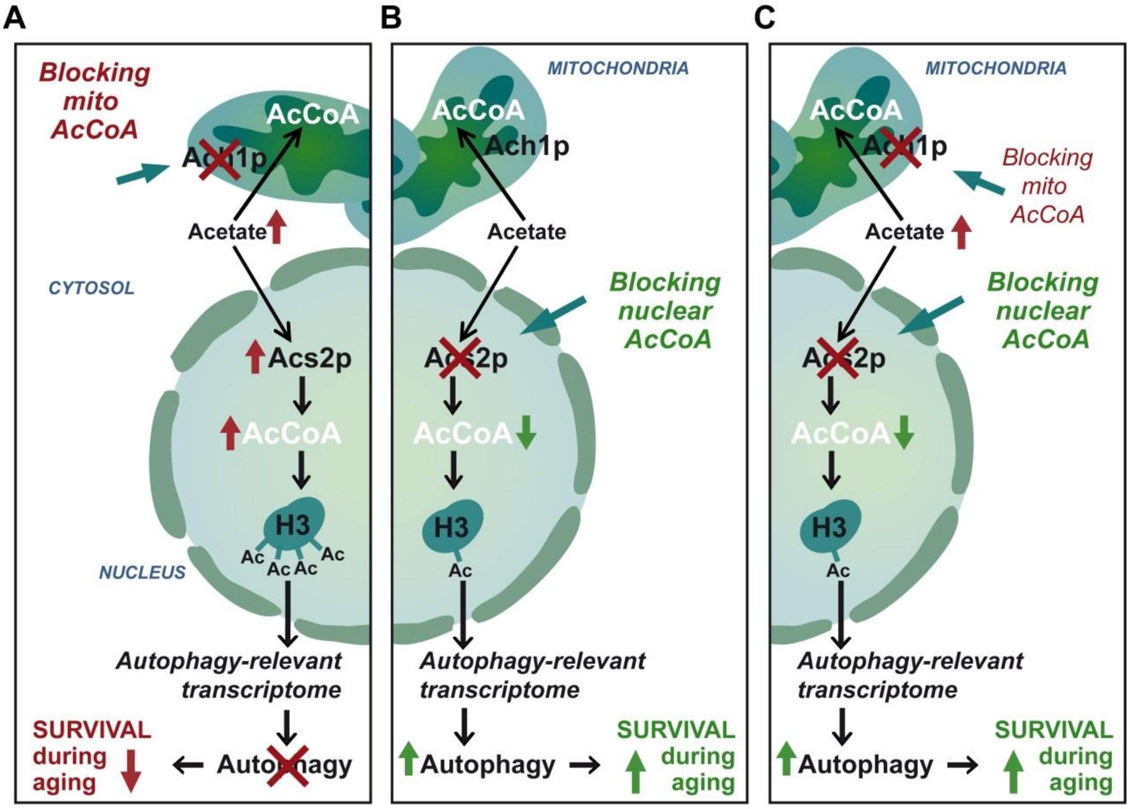


**Figure S7, related to Figures 1 - 7**

***Nucleo-cytosolic Acs2-dependent AcCoA pathway is a dominant suppressor of autophagy.***

(**A-C**) Working hypothesis of how acetate/ Acetyl-CoA metabolism regulates autophagy. (A) Blocking the mitochondrial route of Acetyl-CoA (AcCoA) production by deletion of *ACH1* causes cytosolic accumulation of acetate that in turn activates the nucleo-cytosolic AcCoA synthetase (Acs2p). This leads to hyperacetylation of histone H3 (and also H2A/B, not shown), which modulates the autophagy-relevant transcriptome causing loss of autophagic activity. In contrast, downregulation of Acs2p triggers increased autophagy (B) that dominates over the mitochondrial deficiency of *ACH1* deletion by reversing the defects downstream of Acs2p (C).

**Supplemental Table S1, related to all figures and to Experimental Procedures**

*S. cerevisiae* yeast strains used in this study. For details on construction of strains refer to Supplemental Experimental Procedures and Table S2.

| **Yeast Strain** | **Genotype** | **Origin** | ***Alias*** |
|---|---|---|---|
| BY4741 (WT) | MATa his3Δ-1 leu2Δ-0 met15Δ-0 ura3Δ-0 | *Euroscarf* | - |
| Δ*ach1* | BY4741 ach1Δ::kanMX | *Euroscarf* | - |
| Δ*mpc1* | BY4741 mpc1Δ::kanMX | *Euroscarf* | - |
| Δ*sch9* | BY4741 sch9Δ::HIS3 | This study | TEY1 |
| Δ*ach1*Δ*sch9* | BY4741 ach1Δ::kanMX sch9Δ::HIS3 | This study | TEY2 |
| Δ*ach1*Δ*mpc1* | BY4741 mpc1Δ::kanMX ach1Δ:: hphNT1 | This study | TEY3 |
| Δ*ach1* pATG8-EGFP-ATG8 | BY4741 ach1Δ::kanMX pATG8Δ::natNT2-pATG8-EGFP | This study | TEY4 |
| Δ*mpc1* pATG8-EGFP-ATG8 | BY4741 mpc1Δ::kanMX pATG8Δ::natNT2-pATG8-EGFP | This study | TEY5 |
| Δ*mpc1*Δ*ach1* pATG8-EGFP-ATG8 | BY4741mpc1Δ::kanMX ach1Δ:: hphNT1 pATG8Δ::natNT2-pATG8-EGFP | This study | TEY6 |
| Tet-WT (R1158) | BY4741 URA3::CMV-tTA | *Yeast Tet-Promoters Hughes Collection* (yTHC), *ThermoScientific* | R1158 |
| *Tet-ACS2* | BY4741 URA3::CMV-tTA pACS2Δ::kanR-tet07-TATA | yTHC, *ThermoScientific* | |
| Tet-WT pATG8-EGFP-ATG8 | Tet-WT pATG8Δ::natNT2-pATG8-EGFP | This study | TEY7 |
| *Tet-ACS2* pATG8-EGFP-ATG8 | Tet-ACS2 pATG8Δ::natNT2-pATG8-EGFP | This study | TEY8 |
| Tet-WT Δ*ach1* | Tet-WT ach1Δ::hphNT1 | This study | TEY9 |
| *Tet-ACS2* Δ*ach1* | Tet-ACS2 ach1Δ:: hphNT1 | This study | TEY10 |
| Tet-WT Δ*ach1* pATG8-EGFP-ATG8 | Tet-WT ach1Δ::hphNT1 pATG8Δ::natNT2-pATG8-EGFP | This study | TEY11 |
| *Tet-ACS2* Δ*ach1* pATG8-EGFP-ATG8 | Tet-ACS2 ach1:: hphNT1 pATG8Δ::natNT2-pATG8-EGFP | This study | TEY12 |
| Tet-WT Δ*atg7* | Tet-WT atg7Δ::HIS3 | This study | TEY13 |
| Tet-WT Δ*ach1* Δ*atg7* | Tet-WT ach1Δ::hphNT1 atg7Δ::HIS3 | This study | TEY14 |
| *Tet-ACS2* Δ*atg7* | *Tet-ACS2* atg7Δ::HIS3 | This study | TEY15 |
| *Tet-ACS2* Δ*ach1* Δ*atg7* | *Tet-ACS2* ach1Δ::hphNT1 atg7Δ::HIS3 | This study | TEY16 |
| ATG7-6HA | BY4741 ATG7-6HA-natNT2 | This study | TEY17 |
| Δ*ach1* ATG7-6HA | BY4741 ach1Δ::kanMX ATG7-6HA-natNT2 | This study | TEY18 |

| | | | |
|---|---|---|---|
| Wt-ACS2 | BY4741 acs2::HygMX [pHT112-1, ACS2-CEN-URA3] | Gift from Jef D. Boeke (Takahashi et al., 2006) | YHT651 |
| *Acs2-Ts1* | BY4741 acs2::HygMX [pHT215, acs2-Ts1-CEN-URA3] | Gift from Jef D. Boeke (Takahashi et al., 2006) | YHT652 |
| Tet-WT ATG7-6HA | Tet-WT ATG7-6HA-natNT2 | This study | TEY19 |
| *Tet-ACS2* ATG7-6HA | Tet-ACS2 ATG7-6HA-natNT2 | This study | TEY20 |
| Tet-WT Δ*ach1 ATG7-6HA* | Tet-WT ach1Δ::hphNT1 ATG7-6HA-natNT2 | This study | TEY21 |
| *Tet-ACS2* Δ*ach1 ATG7-6HA* | Tet-ACS2 ach1Δ:: hphNT1 ATG7-6HA-natNT2 | This study | TEY22 |
| Δ*hht1* | BY4741 hht1Δ::kanMX | *Euroscarf* | - |
| Δ*hht1* H3-wt/wt | BY4741 hht1Δ::kanMX HHT2-pRS306-URA3-HHT2 | This study | TEY23 |
| Δ*hht1* H3-KR/KR-1 | BY4741 hht1Δ::kanMX hht2-K14,18R-pRS306-URA3- hht2-K14,18R | This study | TEY24 |
| Δ*hht1* H3-KR/KR-2 | BY4741 hht1Δ::kanMX hht2-K9,14,18R-pRS306-URA3- hht2-K9,14,18R | This study | TEY25 |
| Δ*hht1* H3-KQ/KR-1 | BY4741 hht1Δ::kanMX hht2-K14,18R-pRS306-URA3- hht2-K14,18Q | This study | TEY26 |
| Δ*hht1* H3-KQ/KR-1 | BY4741 hht1Δ::kanMX hht2-K9,14,18R-pRS306-URA3- hht2-K9,14,18Q | This study | TEY27 |
| WT (Vector control) | BY4741 [pESC-HIS] | This study | TEY28 |
| *ACS2* overexpr. | BY4741 [pESC-HIS-ACS2] | This study | TEY29 |
| BY4742 | MATα his3Δ-1 leu2Δ-0 lys2Δ-0 ura3Δ-0 | *Euroscarf* | - |
| BY4742 Δ*ach1* | BY4742 ach1Δ::kanMX | *Euroscarf* | - |

## Supplemental Table S2, related to all Figures and Experimental Procedures

The methods (PCR templates) used for mutant generation (e.g. gene replacement, gene tagging by homologous recombination) are depicted and primers used for cassette generating PCR listed.

| **Target** | **Primers** | **PCR template / Method by** |
|---|---|---|
| *ACH1* deletion | 5’-GCAAAACAAACAACACATTTCTTTTTTTCTTTTTCACATATTGCA CTAAAATGCGTACGCTGCAGGTCGAC-3’ (*S1 Primer*)<br>5´-TCCTTTCTTTTTTTTTGTTAAATACTCATCTCTCGGTTTGCGCACAA ACACTAATCGATGAATTCGAGCTCG-3´ (*S2 Primer*) | pFA6a–hphNT1 / (Janke et al., 2004) |
| Control PCR of *ACH1* deletion | 5’-CATACCACGATCCAAACGAC-3´<br>5’-GTCGACCTGCAGCGTACG-3´ (*S1/S3 reverse*) | |
| N-terminal tagging of *ATG8* with pATG8-EGFP | 5’-CTAATAATTGTAAAGTTGAGAAAATCATAATAAAAATAATTACT AGAGACATGCGTACGCTGCAGGTCGAC-3´ (*S1 primer*)<br>5’-GACTCCGCCTTCCTTTTTTCAAATGGATATTCAGACTTAAATGTA GACTTCATCGATGAATTCTCTGTCG-3´ (*S4 Primer*) | pYM-pATG8[1] / (Janke et al., 2004) |
| Control PCR of *ATG8* tagging | 5’-AGAGAGCTGGTCAACAGAATCC-3´<br>5’-GTCGACCTGCAGCGTACG-3´ (*S1/S3 reverse*) | |
| C-terminal tagging of *ATG7* | 5’-TTACGGAAAGTGGCACCACAATATGTACCAATGCTATTATATGC AAAATAATCGATGAATTCGAGCTCG-3´ (*S2 primer*)<br>5’-TAGGCAACGATGTTTTTGAATGGGAAGATGATGAATCTGATGAG ATTGCTCGTACGCTGCAGGTCGAC-3´ (*S3 primer*) | pYM-17 / (Janke et al., 2004) |
| Control PCR of *ATG7* tagging | 5’-GGCATGGTAATAGAGATGAACAG-3´<br>5’-GTCGACCTGCAGCGTACG-3´ (*S1/S3 reverse*) | |
| *ATG7* deletion | 5’-TAAAGTTCATTATATTTCAACAAATATAAGATAATCAAGAATAA ACAGCTGAAGCTTCGTACGC-3´<br>5’- GAAAGTGGCACCACAATATGTACCAATGCTATTATATGCAAAA TAGCATAGGCCACTAGTGGATC-3´ | pUG27 / (Gueldener et al., 2002) |
| Control PCR of *ATG7* deletion | 5’-TGTACCTCCTGAAGAGCATGA-3´<br>5’-CTGCAGCGAGGAGCCGTAAT-3´ | |
| *SCH9* deletion | 5’-AAAATTTACTCTTTTGGCAACTGTTTATAAGAAGAATAAGTCTG ACAGCTGAAGCTTCGTACGC-3´<br>5’- CAAGAGGAGCGATTGAGAAATCATATTTCGAATCTTCCACTGA CAGCATAGGCCACTAGTGGATC-3´ | pUG27 / (Gueldener et al., 2002) |
| Control PCR of *SCH9* deletion | 5’-CGATAACGGTTCTTTCTGCATAT-3´<br>5’-CTGCAGCGAGGAGCCGTAAT-3´ | |

[1]For details of pYM-pATG8 plasmid construction derived from pYM-N37 (Janke et al., 2004) see Supplemental Experimental Procedures

## Supplemental Table S3, related to Figure 5 and Figure S5

Primers used for quantitative reverse transcription real-time PCR (RT-qPCR).

| Primer specific for… | Sequence |
|---|---|
| *ATG5* (forward) | 5'-TTCGTTGCCACCAACTATCA-3' |
| *ATG5* (reverse) | 5'-AACCAAAACTTTTCCGCTTG -3' |
| *ATG7* (forward) | 5'-GGTAAGCTAGCACCACGTGT-3' |
| *ATG7* (reverse) | 5'-TCCACGGATTGGTCAGCAAT-3' |
| *ATG8* (forward) | 5'-GAGTCGGAGAGGATTGCTGA-3' |
| *ATG8* (reverse) | 5'-CAGCTTTTTCGCAAATCACA-3' |
| *ATG11* (forward) | 5'-GCAAATGTTTACCCCGAATG-3' |
| *ATG11* (reverse) | 5'-GCCATTTGGTCTTCAAGCTC-3' |
| *ATG14* (forward) | 5'-TGGGATGTTGTACTCGATGG-3' |
| *ATG14* (reverse) | 5'-TGCAGGATGTCCTCTTTGTG-3' |

## Supplemental Table S4, related to Figure 5 and Figure S5

Primers used for cloning of pRS306-*hht2-KR/KQ* mutant variants.

| Plasmid | Sequence |
|---|---|
| pRS306-*hht2-K14,18R* | 5'-CACAATGGCCAGAACTAAACAAACAGCTAGAAAATCCACTG GTGGTAGAGCCCCAAGAAGACAATTAG-3' |
| | 5'-CTTAGTCTTCTGGCCAATTTGATATCC-3' |
| pRS306-*hht2-K9,14,18R* | 5'-CACAATGGCCAGAACTAAACAAACAGCTAGAAGATCCACTG GTGGTAGAGCCCCAAGAAGACAATTAG-3' |
| | 5'-CTTAGTCTTCTGGCCAATTTGATATCC-3' |
| pRS306-*hht2-K14,18Q* | 5'-CACAATGGCCAGAACTAAACAAACAGCTAGAAAATCCACTG GTGGTCAGGCCCC AAGACAGCAA TTAG-3' |
| | 5'-CTTAGTCTTCTGGCCAATTTGATATCC-3' |
| pRS306-*hht2-K9,14,18Q* | 5'-CACAATGGCCAGAACTAAACAAACAGCTAGACAGTCCACTG GTGGTCAGGCCCCAAGACAGCAATTAG-3' |
| | 5'-CTTAGTCTTCTGGCCAATTTGATATCC-3' |

## Supplemental Experimental Procedures

### *Yeast Genetics and Culture Conditions*

Experiments were carried out in BY4741 (MATa his3Δ1 leu2Δ0 met15Δ0 ura3Δ0) or BY4742 (MATalpha his3Δ1 leu2Δ0 lys2Δ0 ura3Δ0) and respective mutants as listed in Table S1. Strains were grown at 28 °C on SC medium containing 0.17% yeast nitrogen base (Difco), 0.5% (NH4)2SO4 and 30 mg/l of all amino acids (except 80 mg/l histidine and 200 mg/l leucine), 30 mg/l adenine and 320 mg/l uracil with 2% glucose (SCD). BY4742 cultures were additionally supplemented with 90 mg/l lysine. Aging experiments and tests for cell death markers were performed as described (Büttner et al., 2007; Eisenberg et al., 2009). Briefly, for chronological ageing experiments, cultures were inoculated from fresh overnight cultures to OD600 of 0.1 ($\sim 1 \cdot 10^6$ cells/ml) with the culture volume being 10% of flask volume and aliquots were taken out to perform survival plating at indicated time points with 500 cells plated on YPD agar. If not otherwise stated, representative ageing experiments are shown with at least three independent samples (as indicated) aged at the same time. Experiments have been performed at least three times in total with similar outcome. Tests for apoptotic (Annexin V staining) and necrotic (PI staining) markers as well as markers for oxidative stress (DHE staining) were performed using the Annexin-V-FLUOS Staining Kit (Roche, 11858777001) with a modified protocol suitable for yeast cells as described (Büttner et al., 2007). For quantifications using flow cytometry (BD FACSAria), 30,000 cells were evaluated and analysed with BD FACSDiva software.

Rapamycin (AG Scientific) was supplemented from DMSO stocks (1 mg/ml) to a final concentration of 20 nM prior to inoculation, a condition that did not affect growth of cells during logarithmic phase. Knockdown of *ACS2* was facilitated by supplementing doxycycline hyclate (Sigma, 1 ng/ml final concentration or as indicated) to *tet-ACS2* and respective tet-WT strains (Table S1) prior to inoculation. Acetate (using aqueous stock from potassium acetate adjusted to pH 3 with acetic acid) was administrated to a final concentration of 20 mM to wild type cells as indicated.

For yeast starvation experiments, BY4741 wild type cells harboring either the empty vector control (pESC-HIS) or the pESC-HIS-*ACS2* plasmid were inoculated from fresh overnight cultures to an optical density (OD) of 0.05 at 600 nm ($\sim 1 \times 10^6$ cells/ml) and grown to mid-LOG phase (OD of ~0.2) at 28 °C on Synthetic Complete 2% glucose medium lacking histidine. For induction of Gal10p-driven *ACS2* expression, cells were shifted to SC 2% galactose medium lacking histidine and further incubated for 5 hours. After two washes in double-distilled sterile water, cells were resuspended in Hank's balanced salt solution (HBSS) supplemented with 0.1% galactose at an optical density of 0.75 and incubated for 12 h at 28 °C. For *ACS2* overexpression experiments during chronological aging, cultures were shifted to SC 1.25% galactose / 0.75% glucose (instead of 2 % galactose) medium lacking histidine to facilitate comparable growth of *ACS2* overexpressors as well as empty vector control cells. Notably, *ACS2* overexpressors exhibited considerable growth arrest and increased toxicity on 2% galactose medium compared to 1.25% galactose / 0.75% glucose mixed conditions, where growth was normal under both conditions (not shown).

Yeast plasmid transformation was performed as previously described using lithium acetate method (Gietz and Woods, 2002). Generation of deletion mutants or tagging of genes of interest was performed according to the method described by Janke et al. (Janke et al., 2004). EGFP-*ATG8* fusions were generated using a modified pYM-N37 plasmid (pYM-pATG8) as template. The plasmid pYM-pATG8 was obtained by replacing the Met25 promoter of pYM-N37 with the ATG8 promoter (pATG8) using *SacI* and *XbaI* restriction sites and primers 5'-acgagctcagacagacccaattggtgatg-3' and 5'-actctagaagtctctagtaattattttattatgattttctcaac-3'. The pESC-HIS-ACS2 plasmid was achieved by cloning *ACS2* including its STOP codon amplified from genomic BY4741 wild type DNA (Primers: 5'-acaactagtatgaacaatcaaggaacataaagtag-3' and 5'-atagagctccattacgaaatttttctcatttaag-3') into MCS1 of pESC-HIS vector (Stratagene) using SpeI and SacI restriction enzymes. To generate histone H3 point mutants and corresponding wild type strain (see also Table S1), Δ*hht1* cells (obtained from *Euroscarf*) were transformed with *Eco81I* (Thermo Scientific) cut pRS306-*HHT2* (giving rise to H3-wt/wt, for detailed genotype see Table S1) or with *Eco81I* cut pRS306-*hht2-KR/KQ* mutant variants carrying the respective lysine to arginine (KR) or lysine to glutamine (KQ) point mutations. After transformation and selection on SC glucose plates lacking uracil, cells were plated from YPD overnight cultures on 5-FOA (5-Fluoroorotic Acid, Sigma) plates to select for loss of URA3 - revertants that have crossed out one of the two *HHT2* genes. Single colonies derived from 5-FOA selection were sequenced to identify strains carrying respective *hht2-KR* mutations and re-transformed with *Eco81I* cut pRS306-*hht2-KR/KQ* mutant variants to generate indicated histone H3 variants each containing two copies of mutated *HHT2* (see Table S1). pRS306-HHT2 was generated by cloning the wild type *HHT2* gene including ~500 bp up- and downstream sequence amplified from genomic BY4741 wild type DNA (Primers: 5'-ATCTGAATTCATTAAACGCCTTCTTGTCAGTTG-3' and 5'-ATCTGCGGCCGCCGCTTGATCAGCAGTTCATC-3') into pRS306 using *EcoRI* and *NotI* restriction sites. Mutant variants were subsequently introduced into pRS306-HHT2 using *MscI* restriction enzyme and primers depicted in Table S4.

***Yeast Autophagy Measurements***

To assess ALP activity, BY4741 wild type or Δ*ach1* cells were transformed with and selected for stable insertion of pTN9 HindIII fragment containing genetically engineered form of PHO8 coding for the Pho8 protein lacking its N-terminal transmembrane domain (Pho8pΔN60) (Noda and Klionsky, 2008; Noda et al., 1995). ALP activity was measured similar to Kirisako et al. (Kirisako et al., 1999) using 1 µg total protein assessed by BioRad protein assay (BioRad). In order to correct for intrinsic (background) ALP activity, respective strains without pTN9 insertion have been simultaneously processed and ALP activity subtracted. Yeast strains carrying *pATG8-EGFP-ATG8* fusions were generated according to the method by Janke et al. (Janke et al., 2004) using a modified pYM-N37 plasmid (pYM-pATG8) as template (Table S2). Quantification of micrographs was performed from blinded pictures with 150-300 cells per micrograph and replicate. Autophagic cells were defined as

cells exhibiting clear vacuolar GFP fluorescence and expressed as fraction of viable (PI negative) cells. Immunoblotting followed standard procedures using anti-GFP (Roche, #11814460001), anti-Acs2p (gift from Dr. Jef D. Boeke) and anti-glyceraldehyde-3-phosphate dehydrogenase (GAPDH) antibodies (gift from Dr. Günther Daum).

In order to test for deficiency in Atg7p-dependent Atg8p lipidation machinery ach1 mutant cells were compared with cells lacking ATG1. Atg1 mutants retain a functional Atg8p lipidation system while lacking autophagic activity and thus accumulate EGFP-Atg8p at so-called preautophagosomal structures (Klionsky et al., 2007a). Consistently, atg1 mutants displayed enlarged punctuate structures also after three days of aging, while ach1 mutants lacked these structures almost completely (Figure S5C). This argues in favor of a defect in the Atg8p lipidation machinery upon deletion of ACH1, however, ach1 mutants also failed to mimic the EGFP-Atg8p localization phenotype observed by deletion of ATG7 (Figure S5C). We conclude that the profound autophagy-defect after ACH1 deletion likely involves modulation of more than one ATG transcript and more sophisticated assays will need to be designed that can unveil the precise mechanistic basis of cells that conditionally lost their autophagic capacity only after three days of aging.

***Immunoblotting and Quantification of Histone Acetylation***

Immunoblotting followed standard protocols with antibodies specific for Acs2p (gift from Dr. Jef D. Boeke), yeast GAPDH (gift from Dr. Günther Daum), acetylated lysine (#9441, Cell Signaling), HA (Sigma) and GFP (#11814460001, Roche). Histone acetylation was detected by means of acetylated histone-site-specific antibodies (#07-352, #07-353 and #07-354, Upstate, Millipore; ab15823 and ab46983, Abcam; #39167, Active Motif) and compared to antibodies recognizing total histone H3 and H4 (ab1791 and ab7311, respectively, Abcam) serving as a loading control. Acid extracts of yeast cells (Kao and Osley, 2003), immunoblot procedures as well as quantification (expressed as ratios of acetylated lysine per total histone signals) using serial dilutions to determine the linear range of histone detection was performed essentially as described (Eisenberg et al., 2009).

***Immunoprecipitation of Acetylated Proteins***

For enrichment of acetylated proteins, immunoprecipitation (IP) of wild type or Δ*ach1* protein extracts was either performed separately for immunoblotting using unlabeled cell cultures or for subsequent analyses with mass spectrometry (MS) using stable-isotope labeled cell cultures (SILAC) from yeast (Lys0, Lys4 or Lys8 labeled) pooled in equal amounts of protein before IP. Immunoblotting of Ac-Lys IP eluates using pan-Acetyl-lysine antibodies confirmed that the differences in Ac-Lys signal intensities of WT and Δ*ach1* extracts were readily present after IP (see main manuscript Figure 4C, *Ac-Lys IP*).

Approximately 3 x $10^9$ cells (20 ml aged cell culture) were washed once with 5 ml buffer R (50 mM Tris/HCl pH 7.4, 1% Triton X-100, 150 mM NaCl, 1 mM EDTA) and the cell pellet snap frozen in liquid nitrogen. Cells were resuspended in buffer R+, which is buffer R supplemented with 1x Complete® protease inhibitor cocktail (Roche), 30 mM trichostatin A (Sigma) and 2 µM nicotinamid (Sigma), and disrupted by vortexing in the presence of glass

beads (0.45 - 0.5 mm, VWR) for 2x 2 min at 4 °C. Protein concentration was determined using BioRad protein assay (BioRad) and adjusted to 1 mg/ml with buffer R+. This crude lysate (*Input*) was immediately subjected to pre-clearance from proteins unspecifically binding to beads (yielding the *Pre-lysate*) using 50 µl PureProteome Protein G Magnetic Beads suspension (Millipore) per ml of Input. Pre-clearance was performed for 1 hour at 4 °C. Beads have been prepared according to the manufactures' instructions. The Pre-lysate of SILAC-mixed samples was either snap frozen for later mass spectrometric analyses (to detect general protein abundance) or immediately used for IP.

For IP using 250 µl (unlabeled) or 1500 µl (SILAC-labeled) total volume, pan-Acetyl lysine antibody binding (1:100, Cell Signaling; an equal mix of Ac-K (#9441) and Ac-K2 (#9814) antibodies was used) was performed at indicated protein concentrations (see Figure 3) and incubated for 4 hours at 4 °C with gentle rotation. Antibody capture was performed at room temperature for 10 min using 50 µl magnetic beads per ml sample. Samples were washed five times with ice-cold buffer R and purified proteins eluted in 1x *Laemmli* for 10 min at 85 °C.

***MS Sample Preparation***

Samples (Pre-lysates or IP eluates) were reduced with 1 mM DTT (Sigma-Aldrich) for 5 min at 95 °C and alkylated using 5.5 mM iodoacetamide (Sigma-Aldrich) for 30 min at 25 °C. Protein mixtures were separated by SDS-PAGE using 4-12% Bis-Tris mini gradient gels (NuPAGE, Invitrogen). The gel lanes were cut into 10 equal slices, which were in-gel digested with LysC (Wako, Richmond, U.S.A.) (Shevchenko et al., 2007), and the resulting peptide mixtures were processed on STAGE tipps as described (Rappsilber et al., 2007).

***MS Measurement***

Mass spectrometric measurements were performed on LTQ Orbitrap XL mass spectrometer (Thermo Fisher Scientific, Bremen, Germany) coupled to an Agilent 1200 nanoflow-HPLC (Agilent Technologies GmbH, Waldbronn, Germany). HPLC-column tips (fused silica) with 75 µm inner diameter (New Objective, Woburn, MA, USA) were self-packed with Reprosil-Pur 120 ODS-3 (Dr. Maisch, Ammerbuch, Germany) to a length of 20 cm. Samples were applied directly onto the column without pre-column. A gradient of A [0.5% acetic acid (high purity, LGC Promochem, Wesel, Germany) in water (HPLC gradient grade, Mallinckrodt Baker B.V., Deventer, Netherlands)] and B [0.5% acetic acid in 80% ACN (LC-MS grade, Wako, Germany) in water] with increasing organic proportion was used for peptide separation (loading of sample with 2% B; separation ramp: from 10% to 30% B within 80 min). The flow rate was 250 nl/min and for sample application 500 nl/min. The mass spectrometer was operated in the data-dependent mode and switched automatically between MS (max. of 1 $x10^6$ ions) and MS/MS. Each MS scan was followed by a maximum of five MS/MS scans in the linear ion trap using normalized collision energy of 35% and a target value of 5,000. Parent ions with a charge state from z = 1 and unassigned charge states were excluded for fragmentation. The mass range for MS was m/z = 370 to 2,000. The resolution was set to 60.000. Mass-spectrometric parameters were as follows: spray voltage 2.3 kV; no sheath and auxiliary gas flow; ion-transfer tube temperature 125°C.

### *Identification of proteins and protein ratio assignment using MaxQuant*

The MS raw data files were uploaded into the MaxQuant software version 1.2.2.5 (Cox and Mann, 2008) and searched with the Andromeda search engine (Cox et al., 2011) against the yeast UniProt database (release 2012_11). Carbamidomethylcysteine was set as fixed modification; lysine acetylation, methionine oxidation and protein amino-terminal acetylation were set as variable modifications. Triple SILAC were chosen as quantitation mode. Note that triple SILAC was chosen, since in addition to the extracts from aged (day 3) WT and *ach1* mutant cells, extracts of young WT cells (day 1) were co-processed and analyzed by MS. However, data presented in this manuscript solely refer to the comparison of aged WT and ach1 mutant conditions. Two miss cleavages were allowed, enzyme specificity was LysC/P+DP, and the MS/MS tolerance was set to 0.5 Da. The average mass precision of identified peptides was in general less than 1 ppm after recalibration. Peptide lists were further used by MaxQuant to identify and relatively quantify proteins using the following parameters: peptide, and protein false discovery rates (FDR) were set to 0.01, maximum peptide posterior error probability (PEP) was set to 1, minimum peptide length was set to 6, minimum number of peptides for identification and quantitation of proteins was set to one unique peptide, minimum ratio count was set to two, and identified proteins have been re-quantified. The “match-between-run” option (2 min) was used. Mean SILAC ratios of all identified proteins were close to 1, validating labeling and equal mixture of respective SILAC conditions.

### *Acetyl-CoA-Synthetase Activity*

To detect Acetyl-CoA synthetase (ACS) activity, we employed a published enzyme assay, in which generation of AcCoA from acetate and CoA is coupled in a two-step reaction to generation of NADH by malic acid dehydrogenase (Berg et al., 1996).  In principle, this assay does not distinguish the activities of Acs1p and Acs2p. In order to specifically address the activity of Acs2p, we took advantage of the acs1 mutant background, comparing strains with (Δacs1Δach1) or without (Δacs1) additional deletion of ACH1. Of note, the combined deletion of ACS1 and ACH1 had no major impact on (i) survival, (ii) frequency of PI positive (dead) cells or (iii) on the accumulation of extracellular acetate (Figure S3J-L).

Briefly, ~$5*10^8$ cells were subjected to glass bead disruption and protein concentration determined using BioRad Protein Assay (BioRad). Generation of NADH was assessed from 10 µg total protein by measuring the absorbance (optical density, OD) at 340 nm (TECAN plate reader). The reaction was recorded over 20 min in 45 sec intervals with 100 µl assay volume. Slopes determined by linear regression were used to calculate relative ACS activity expressed as OD340 per min and µg protein.

### *Quantitative reverse-transcriptase PCR*

1.0 to $1.5*10^8$ cells aged to day 3 were harvested and total RNA was extracted using the glass bead protocol of Qiagen RNeasy Kit (Qiagen), followed by DNase I digest using RNAse-free DNAse I (Fermentas, 1 u/µg RNA) in the presence of RNAseOUT® RNAse Inhibitor (Life

Tech, Invitrogen). Reverse transcription was performed with 500 ng RNA using Random Hexamer Primers (Life Tech, Invitrogen) and Superscript III Reverse Transcriptase (Life Tech, Invitrogen) according to the enzyme manual. Quantitative PCR using appropriately diluted samples (as determined by standard curves using dilution series of sample mixes) was done with an ABI StepOnePlus® using SYBR®Select Master Mix (Life Tech, Invitrogen) and a final concentration of 200 µM of each primer (see Table S3 for primer sequences). PCR efficiencies resulted to 90 - 110% for all PCR reactions. Target mRNA quantification was calculated by relative ΔCt comparison (ΔΔCt-method) using 18S rRNA as an internal standard and subsequently normalized to signals from wild type cells.

***Drosophila life span analyses and brain immunofluorescence***

In Fly Stocks and Rearing Conditions:

UAS-RNAi lines for Acetyl-CoA Synthetase (P{TRiP.HMS02314}attP2) and EGFP (P{VALIUM20-EGFP.shRNA.1}attP2) were obtained from Bloomington Drosophila Stock Center. UAS-RNAi for EGFP serves as a background matched control here. Isogenised pan-neural driver line APPL-Gal4 was used to drive RNAi expression in fly brains.

All fly strains used in the experiments were reared under standard laboratory conditions in big fly food bottles (containing 3-4 cm of fly food) with overlapping generations (25 C with 12:12 hour light:dark cycle at constant humidity) . Fly food was prepared according to Bloomington media recipe (www.flystocks.bio.indiana.edu/Fly_Work/media-recipes/media-recipes.html). For all experiments, respective crosses were set and allowed to mate on normal food at above-mentioned conditions. For all experiments, flies were reared at standard larval density and eclosing adults were collected once in a day, as a result specific age indicated will be day 24hours. Flies used in all experiments are F1 progeny.

Survival analyses during aging and life span calculation:

Following eclosion, flies were collected, transferred to fresh bottles and left to mate for ~ 24 hours. Following mating, 2-day old flies were anesthetized, using mild CO2 and separated for males and females. Flies were then randomly allocated to small fly culture bottles (containing 3-4mm food) and allowed to age at 25 C, constant humidity, on a 12:12 hour light:dark cycle. Flies were transferred onto new food every 2nd day and scored for deaths while transferring. Flies that flew away while transferring or were sticking to the food were taken under censored observation. Initial cohort sizes were calculated as the summed death and censor observations over all ages.

Immunohistochemistry, Confocal Imaging and Quantification:

Adult brains from male flies were dissected in HL3 on ice and immediately fixed in cold 4% PFA for 20min at room temperature. After fixation, the samples were then incubated in 1% PBT (PBS containing 1% Tween) for 20 min and pre-incubated in 0.3% PBT (PBS containing 0.3% Tween) with 10% NGS for 2 hours at room temperature. For primary antibody treatment, samples were incubated in 0.3% PBT containing 5% NGS and the primary antibodies for 48 hours at room temperature. After primary antibody incubation, brains were

washed in 0.3% PBT, 2 times for 20 min at room temperature, then overnight at 4 C. All samples were then washed in 0.3% PBT, 2 times for 20 min at room temperature. All samples were then incubated in 0.3% PBT with 5% NGS containing the secondary antibodies for 24 h at room temperature. Brains were washed for 2 times for 20 min each with 0.3% PBT at room temperature, then overnight at 4 C. All samples were again washed with 0.3% PBT, 2 times for 20 min and then mounted in Vectashield® for 24h at -20 C before confocal scanning (Vector Laboratories). Antibodies were used at the following dilutions: rabbit Ref(2)P (1:5000) and goat-α-rabbit Cy5 (1:400).
Image stacks of specimens were imaged on a Leica TCS SP8 confocal microscope (Leica Microsystems) using a 20X oil objective for whole brain imaging. Images were quantified using Fiji software (Fiji.sc/Fiji). Briefly, confocal stacks were merged into a single plane by using the maximum projection function. Subsequently, region of central brain was manually selected (using the free hand function) and fluorescence intensity (arbitrary units) were measured and normalized to the area of the central brain for each brain.

***Supplemental Statistical Analyses***

For all experiments presented in Supplemental Information, one-way analyses of variance (ANOVA) and planned comparisons of means were made using Bonferroni test. Error bars represent standard error of the mean (SEM) of biological replicates as indicated. Data obtained in *Drosophila* experiments were analyzed with Prism (GraphPad Software).

## Supplemental References

Büttner, S., Eisenberg, T., Carmona-Gutierrez, D., Ruli, D., Knauer, H., Ruckenstuhl, C., Sigrist, C., Wissing, S., Kollroser, M., Fröhlich, K.-U., et al. (2007). Endonuclease G regulates budding yeast life and death. Mol. Cell *25*, 233–246.

Cox, J., and Mann, M. (2008). MaxQuant enables high peptide identification rates, individualized p.p.b.-range mass accuracies and proteome-wide protein quantification. Nat. Biotechnol. *26,* 1367–1372.

Fabrizio, P., Gattazzo, C., Battistella, L., Wei, M., Cheng, C., McGrew, K., and Longo, V.D. (2005). Sir2 blocks extreme life-span extension. Cell *123*, 655–667.

Gietz, R.D., and Woods, R.A. (2002). Transformation of yeast by lithium acetate/single-stranded carrier DNA/polyethylene glycol method. Methods Enzymol. *350*, 87–96.

Gueldener, U., Heinisch, J., Koehler, G.J., Voss, D., and Hegemann, J.H. (2002). A second set of loxP marker cassettes for Cre-mediated multiple gene knockouts in budding yeast. Nucleic Acids Res. *30*, e23.

Herker, E., Jungwirth, H., Lehmann, K.A., Maldener, C., Fröhlich, K.-U., Wissing, S., Büttner, S., Fehr, M., Sigrist, S., and Madeo, F. (2004). Chronological aging leads to apoptosis in yeast. J. Cell Biol. *164*, 501–507.

Janke, C., Magiera, M.M., Rathfelder, N., Taxis, C., Reber, S., Maekawa, H., Moreno-Borchart, A., Doenges, G., Schwob, E., Schiebel, E., et al. (2004). A versatile toolbox for PCR-based tagging of yeast genes: new fluorescent proteins, more markers and promoter substitution cassettes. Yeast *21*, 947–962.

Kao, C.-F., and Osley, M.A. (2003). In vivo assays to study histone ubiquitylation. Methods San Diego Calif *31*, 59–66.

Noda, T., Matsuura, A., Wada, Y., and Ohsumi, Y. (1995). Novel System for Monitoring Autophagy in the Yeast Saccharomyces cerevisiae. Biochem. Biophys. Res. Commun. *210*, 126–132.

Rappsilber, J., Mann, M., and Ishihama, Y. (2007). Protocol for micro-purification, enrichment, pre-fractionation and storage of peptides for proteomics using StageTips. Nat. Protoc. *2*, 1896–1906.

Shevchenko, A., Tomas, H., Havli|[sbreve]|, J., Olsen, J.V., and Mann, M. (2007). In-gel digestion for mass spectrometric characterization of proteins and proteomes. Nat. Protoc. *1*, 2856–2860.
